# Modulating the local microenvironment over isolated nickel sites through first-shell coordination to regulate the reaction pathway of CO_2_ electroreduction

**DOI:** 10.1093/nsr/nwaf173

**Published:** 2025-05-06

**Authors:** Yan Kong, Xinmei Jia, Xiaoyan Chai, Zhi Chen, Chunyan Shang, Xingxing Jiang, Huizhu Cai, Lingyan Jing, Qi Hu, Hengpan Yang, Xue Zhang, Chuanxin He

**Affiliations:** College of Chemistry and Environmental Engineering, Shenzhen University, Shenzhen 518060, China; Department of Chemical Physics, University of Science and Technology of China, Hefei 23002, China; College of Chemistry and Environmental Engineering, Shenzhen University, Shenzhen 518060, China; Department of Chemical Physics, University of Science and Technology of China, Hefei 23002, China; College of Chemistry and Environmental Engineering, Shenzhen University, Shenzhen 518060, China; College of Chemistry and Environmental Engineering, Shenzhen University, Shenzhen 518060, China; College of Chemistry and Environmental Engineering, Shenzhen University, Shenzhen 518060, China; College of Chemistry and Environmental Engineering, Shenzhen University, Shenzhen 518060, China; College of Chemistry and Environmental Engineering, Shenzhen University, Shenzhen 518060, China; College of Chemistry and Environmental Engineering, Shenzhen University, Shenzhen 518060, China; College of Chemistry and Environmental Engineering, Shenzhen University, Shenzhen 518060, China; College of Chemistry and Environmental Engineering, Shenzhen University, Shenzhen 518060, China; College of Chemistry and Environmental Engineering, Shenzhen University, Shenzhen 518060, China; College of Chemistry and Environmental Engineering, Shenzhen University, Shenzhen 518060, China

**Keywords:** Ni single-atom catalysts, first-shell coordination, methane, local microenvironment modulation, CO_2_ electroreduction

## Abstract

Manipulating the local microenvironments of single-atom catalysts is crucial for the product selectivity of CO_2_ electroreduction. Although theoretical research suggests that modifying the coordination structure of isolated Ni sites can promote the reduction of CO_2_ to CH_4_, there is still no experimental evidence to date. Herein, by regulating the coordination shell of boron (B) surrounding the Ni central atom, we have achieved the transformation of the reduction product from CO to CH_4_. *In situ* techniques and density functional theory calculations reveal that B coordination in the second shell of the Ni–N–C motifs (Ni–N_4_–B/C) facilitates CO formation whereas incorporating B into the first shell (Ni–N_3_B_1_/C) significantly tunes the electronic structure of the Ni atoms, leading to electron delocalization, which enhances the *CO intermediate adsorption strength and makes CH_4_ the dominant product. This study marks the experimental realization of electrochemical CO_2_-to-CH_4_ conversion at isolated Ni sites and underscores the importance of local coordination environment regulation in steering the reaction pathways of single-atom catalysts.

## Teaser

Modulating the local microenvironment around single-atom sites is anticipated to steer the reaction pathway in CO_2_ reduction reactions. By specifically altering the coordination shell of boron around the Ni atoms, a Ni single-atom catalyst achieved a product transformation from CO to CH_4_, with a CH_4_ Faradaic efficiency of >50%.

## INTRODUCTION

The continuous rise in atmospheric CO_2_ concentrations has captured worldwide attention for decades [1,2]. To mitigate carbon emissions and achieve carbon neutrality, the electrocatalytic CO_2_ reduction reaction (CO_2_RR) in conjunction with clean energy sources has emerged as a promising strategy for upgrading CO_2_ into energy-dense hydrocarbons [3–6]. However, the CO_2_RR process is inherently complex and involves multiple proton-coupled electron-transfer (PCET) steps as well as sophisticated intermediates, which results in a variety of products, and thus poor selectivity for a single desired product [7–9]. To address this issue, it is imperative to tune the reaction pathway carefully.

Previous research has highlighted the pivotal function of active sites and their local microenvironment modulation in steering reaction pathways [10,11]. In this context, single-atom catalysts (SACs), with ultra-high atomic utilization, well-defined geometrical configurations and tunable coordination environments, are acknowledged as exemplary model systems for adjusting reaction pathways and elucidating reaction mechanisms [12–14]. Especially, Ni SACs demonstrate the significant potential for the electrocatalysis of CO_2_ to CO due to their suitable adsorption and conversion for *COOH and *CO intermediates compared with other metal SACs [15–17]. Nevertheless, the elementary Ni–N_4_ configuration, characterized by highly symmetric geometries and electronic structures, typically hinders axial CO_2_ adsorption and activation, leading to high kinetic overpotentials [18,19].

Modulating the local microenvironment around the Ni sites can effectively address these challenges. Specifically, lowering the coordination number of Ni centers (NiN*_x_, x* < 4) [20–22], introducing heteroatoms (e.g. P, S, O, F) into various coordination shells [23–30] or using a combination of both strategies [31] can optimize the electronic structure of Ni atoms and alter the adsorption strength of key intermediates, ultimately affecting the reaction kinetics and selectivity for CO [32,33]. Notably, these strategies have not led to the generation of hydrocarbons such as CH_4_, primarily because these modifications have been tailored to enhance the adsorption of *COOH or accelerate *CO desorption. In contrast, the formation of CH_4_ involves a more intricate PCET mechanism. This process frequently necessitates strong *CO adsorption and adequate accessibility of protons in the reaction environment [34]. Consequently, this imposes greater demands on the design of active sites. Theoretical studies revealed that the introduction of B atoms into the first coordination shell of Ni sites can markedly enhance the electron density around the central metal, more significantly than other heteroatoms (C, N, O, S and P) [35,36]. This enhancement facilitates stronger interactions with *CO and further hydrogenation to *CHO, thereby promoting efficient CH_4_ production. However, this theoretical prediction has yet to be experimentally confirmed.

To elucidate the impact of local coordination environment modulation on reaction pathways, we synthesized a set of B-coordinated Ni single-atom electrocatalysts. Our findings revealed distinct product selectivity, depending on the B atom coordination shell: B positioned in the second shell facilitated only CO production whereas B in the first shell enabled CH_4_ formation. This regulation alters the reaction pathway, thereby enabling the product conversion from CO to CH_4_. The Ni–N_3_B_1_/C catalyst exhibited a peak Faradaic efficiency of 55.4% for CH_4_ at a total current density of 600 mA cm^−2^. Investigations of the mechanism and density functional theory (DFT) calculations unveiled that incorporating B into the first coordination shell effectively modulated the electronic structure of the Ni sites and elevated the d-band center. This alteration results in the delocalization of the electron distribution around the Ni atoms, thereby enhancing the Ni–C bonding interaction and promoting further hydrogenation to *CHO intermediates. Moreover, Lewis acid B sites demonstrated exceptional water-dissociation capabilities, increasing the availability of protons that were essential for CH_4_ production. This work is the first to achieve significant conversion of CO_2_ to CH_4_ by using Ni SACs and provides valuable insights into the potential of modifying intrinsic activity through tuning the local microenvironment of SACs.

## RESULTS AND DISCUSSION

### Synthesis and characterization of catalysts

**Figure 1.**
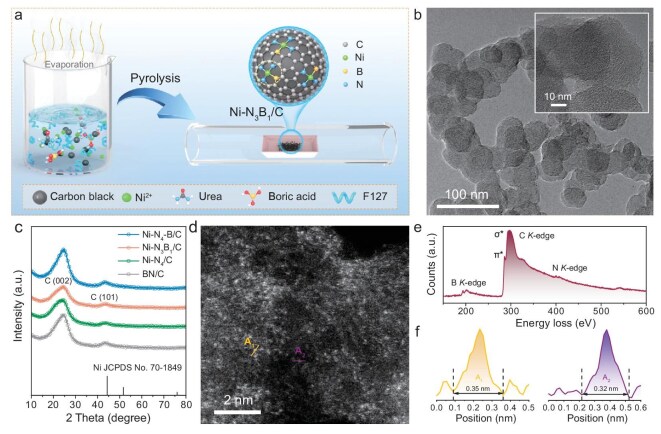
Synthesis and structural characterization of the Ni–N_3_B_1_/C electrocatalysts. (a) Schematic diagram of the typical preparation procedure for Ni–N_3_B_1_/C. (b) TEM image of Ni–N_3_B_1_/C. Insert in (b) displays the HRTEM image. (c) XRD patterns of Ni SACs and BN/C. (d) Aberration-corrected HAADF-STEM image of Ni–N_3_B_1_/C. (e) EELS analysis of Ni–N_3_B_1_/C. (f) The size of isolated Ni atoms based on points A_1_ and A_2_ in (d).

The Ni single-atom catalyst simultaneously coordinated with both B and N atoms was synthesized through a refined, continuous two-step approach [37]. An illustration of the preparation procedure for the catalyst is described in Fig. 1a. Initially, a homogeneous precursor containing N, B and Ni species was prepared via solvent evaporation. Subsequently, the above precursor underwent a carbonization process at 800°C under a nitrogen atmosphere, resulting in the formation of the B, N-coordinated Ni single-atom catalyst, denoted as Ni–N_3_B_1_/C. Additionally, to shed light on the influence of the local coordination environment around the central Ni metal, additional reference materials such as Ni–N_4_/C and Ni–N_4_–B/C, were also synthesized by following a similar avenue. Inductively coupled plasma-optical emission spectroscopy analysis revealed the Ni content to be 1.24 wt% for Ni–N_4_/C, 1.18 wt% for Ni–N_3_B_1_/C and 1.61 wt% for Ni–N_4_–B/C, respectively. The similar Ni contents in the three catalysts indicate that the performance difference is determined by the local coordination environment, rather than metal loading.

Scanning electron microscopy (SEM) and transmission electron microscopy (TEM) images reveal that all the as-prepared Ni SACs maintain a spherical morphology with a relatively uniform particulate size distribution (Figs S1 and S2). Further observation by using high-resolution transmission electron microscopy (HRTEM) demonstrates the absence of metal nanoparticles or clusters through the carbon substrate (Fig. 1b). The corresponding energy-dispersive X-ray spectroscopy mapping images exhibit a homogeneous dispersion of C, N, B and Ni elements in these samples (Figs S3–S5). Notably, X-ray diffraction (XRD) patterns of the catalysts, presented in Fig. 1c, exhibit only the fingerprint peaks associated with graphitic carbon. No other characteristic peaks, such as those assigned to Ni, NiO*_x_* or NiB*_x_*, are discerned, indicating the formation of highly dispersed Ni species across the entire architecture. Likewise, as illustrated in Fig. S6, the Raman spectra also identify only two vibrational bands aligned with carbon materials. To ascertain the distribution of Ni species in Ni–N_3_B_1_/C, we harnessed aberration-corrected high-angle annular dark-field scanning transmission electron microscopy (HAADF-STEM). As depicted in Fig. 1d, the carbon framework is populated by randomly dispersed scattered bright dots. These isolated bright dots, with an approximate size of 0.33 nm, represent the single Ni atoms (Fig. 1f). Electron energy loss spectroscopy (EELS), as shown in Fig. 1e, corroborates the exclusive coordination of these isolated Ni atoms adjacent to B and N atoms.

**Figure 2.**
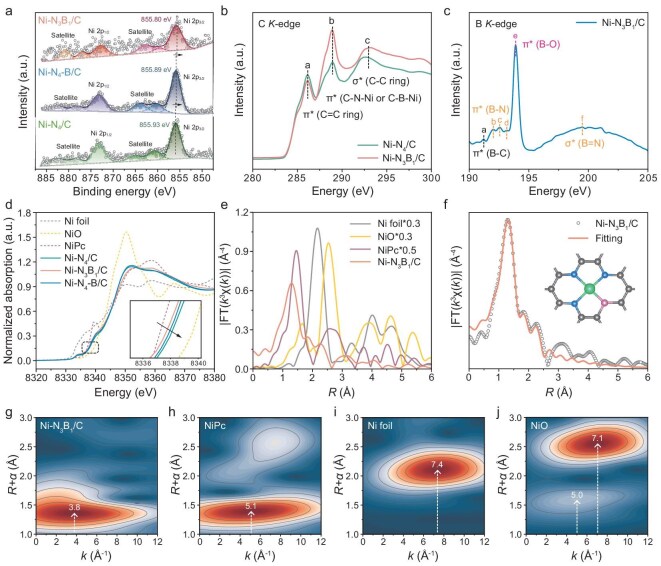
Electronic structure and coordination environment analysis of Ni SACs. (a) High-resolution Ni 2*p* spectra of Ni–N_4_/C, Ni–N_3_B_1_/C and Ni–N_4_–B/C. (b) C *K*-edge XANES spectra of Ni–N_4_/C and Ni–N_3_B_1_/C. (c) B *K*-edge XANES spectra of Ni–N_3_B_1_/C. (d) Normalized Ni *K*-edge XANES spectra of Ni SACs and references. The inset shows the locally enlarged spectra. (e) Fourier-transformed EXAFS spectra of Ni–N_3_B_1_/C and references. (f) The corresponding EXAFS fitting curves in the *R*-space of Ni–N_3_B_1_/C. Wavelet transform EXAFS plots of (g) Ni–N_3_B_1_/C, (h) NiPc, (i) Ni foil and (j) NiO.

The chemical states and elemental composition were investigated by using X-ray photoelectron spectroscopy (XPS). The high-resolution Ni 2*p* spectra of Ni–N_3_B_1_/C reveal a slight negative shift in the binding energy of Ni 2*p*_3/2_ (855.80 eV) compared with those of Ni–N_4_–B/C (855.89 eV) and Ni–N_4_/C (855.93 eV) (Fig. 2a), indicative of the lowest oxidation state of Ni in Ni–N_3_B_1_/C. This is attributed to the incorporation of a less-electronegative heteroatom, boron, in its first coordination shell. Additional component contributions related to B–C and B–N are observed in both the Ni–N_3_B_1_/C and Ni–N_4_–B/C catalysts (Figs S7–S9), further demonstrating the successful integration of B atoms within the nitrogen-doped carbon substrate.

To further elucidate the electronic structure and coordination environment of Ni SACs, we employed X-ray absorption near-edge structure (XANES) spectra to probe the configurations of C, N and B elements. The C *K*-edge spectra (Fig. 2b) reveal three distinct peaks at 286.11 eV (Peak a), 288.85 eV (Peak b) and 293.09 eV (Peak c), which is attributed to the dipole transition of C 1*s* orbital electrons into π* (C=C ring), π* (C–N/B–Ni) and σ* (C–C ring) anti-bonding orbitals, respectively [38–40]. It is worth mentioning that Ni–N_3_B_1_/C presents diminished peak intensity for C=C while showing augmented peak intensity for C–N/B–Ni in comparison with Ni–N_4_/C. This result indicates that the incorporation of B atoms to some extent modifies the electronic structure and chemical bond within the composites, leading to the formation of more C–N/B bonds in the carbon matrix [41]. Additionally, the B *K*-edge spectrum for Ni–N_3_B_1_/C exhibits six characteristic peaks, assigned to B–C, B–N, B–O configurations π* state and B=N configuration σ* state, respectively (Fig. 2c) [42,43]. The N *K*-edge spectra of both Ni–N_4_/C and Ni–N_3_B_1_/C demonstrate the presence of multiple types of N species, including pyridinic-N (Peak a), pyrrolic-N (Peak b) and graphitic-N (Peak c), along with a broadened peak (Peak d) originating from the transition of N 1*s* orbital into σ* orbital (Fig. S10) [44,45], corresponding well with the XPS results. These observations strongly confirm the significant interactions between carbon, nitrogen and boron atoms within the catalyst architecture.

The normalized Ni *K*-edge XANES spectra in Fig. 2d reveal that the absorption edges of Ni–N_4_/C, Ni–N_3_B_1_/C and Ni–N_4_–B/C are situated between the Ni foil and NiO references, indicating that the average oxidation state of Ni in these three samples falls between Ni^0^ and Ni^2+^. Notably, the sequence of the absorption edges is as follows: Ni–N_3_B_1_/C < Ni–N_4_–B/C < Ni–N_4_/C, suggesting that the incorporation of B atoms results in less electron deficiency at the Ni sites compared with N atoms, which can be attributed to the weaker electronegativity of B. Furthermore, the pre-edge absorption feature (1*s*→3*d*/4*p*), which is highly sensitive to symmetry, provides more insight into the local geometrical structure surrounding the Ni centers. The 1*s*→3*d* transition, typically symmetry-forbidden for centrosymmetric point groups, can gain intensity in geometries allowing *p–d* mixing [46]. Herein, a sharper pre-edge peak at 8333.9 eV is observed for Ni–N_3_B_1_/C in comparison with Ni–N_4_/C, Ni–N_4_–B/C and NiPc, indicative of deviated quadrilateral NiN_3_B moieties with decreased coordination symmetry. Overall, B doping enhances the mixing of the 4*p* character into the 3*d* orbitals, leading to a stronger pre-edge absorption peak, which further supports the asymmetric incorporation of B and N atoms around the Ni centers.

The Fourier-transformed extended X-ray absorption fine structure (FT-EXAFS) spectra (Fig. 2e and Fig. S11) of Ni–N_4_/C, Ni–N_3_B_1_/C and Ni–N_4_–B/C all exhibit a dominant peak at ∼1.36 Å, corresponding to the Ni–N/Ni–B coordination in the first shell, which more closely resembles the Ni–N scattering (∼1.45 Å) observed in NiPc. No typical peak for the Ni–Ni contribution at ∼2.2 Å is noticed, confirming the absence of Ni particles or clusters and the presence of isolated Ni sites in these samples. Subsequently, quantitative EXAFS fitting was performed to investigate the fine differences in the coordination configurations of the Ni atoms on these catalysts. The detailed structural parameters are summarized in Table S1. For the Ni–N_4_/C catalyst, the fitting includes Ni–N and Ni–C scattering paths with a Ni–N coordination number of 3.9, indicating that the Ni atom is coordinated with four N atoms in the first shell (Fig. S12a). Meanwhile, the EXAFS fitting curves of Ni–N_3_B_1_/C unveil three scattering pathways: Ni–N, Ni–B and Ni–C, with optimal fitting results showing that the Ni atom is directly linked to three N atoms and one B atom (Fig. 2f). In contrast, for Ni–N_4_–B/C, along with the Ni–N path with a coordination number of 3.6, a shoulder peak at 2.72 Å is discerned, possibly ascribed to the Ni–B/Ni–C scattering in the second shell (Fig. S12b). Moreover, the *k*-space EXAFS fitting results are displayed in Fig. S13, providing a complementary perspective on the structural configuration. The wavelet transform (WT) of the Ni *K*-edge oscillations was further implemented to discriminate distinct backscattering atoms, as they can integrate information from both *k*-space and *R*-space. The Ni SACs exhibit a peak at ∼4.0 Å^−1^, which is entirely different from the Ni–Ni bonds (7.4 Å^−1^) observed in the Ni foil (Fig. 2g–j and Fig. S14), further substantiating the atomically dispersed Ni species in Ni–N_4_/C, Ni–N_3_B_1_/C and Ni–N_4_–B/C. It is noteworthy that slight WT peak shifts in the *k*-space of Ni–N_3_B_1_/C and Ni–N_4_–B/C compared with Ni–N_4_/C indicate discrepancies in their neighboring coordination environments.

### Electrochemical CO_2_RR performance

To investigate the impact of different atomic coordination configurations around the Ni sites on the selectivity for CO_2_RR products, we conducted a series of systematic electrochemical tests. Given the poor solubility and sluggish mass transfer of CO_2_ in solution, the performances of catalysts were assessed by using a three-electrode flow cell reactor with 1 M KOH as the electrolyte (Fig. S15). Nuclear magnetic resonance (NMR) spectroscopy and gas chromatography (GC) were leveraged to quantify the liquid- and gas-phase products, respectively (Figs S16 and S17).

**Figure 3.**
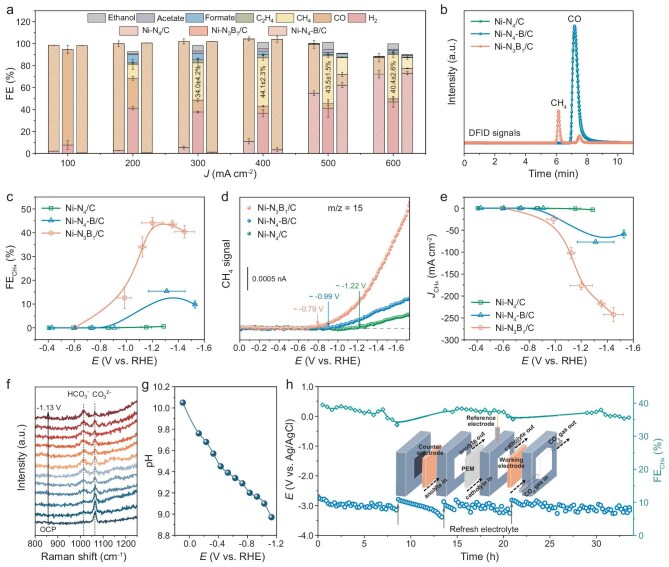
Electrochemical CO_2_-reduction performance. (a) Faradaic efficiencies for the combined products on the prepared catalysts. (b) CH_4_ and CO signals detected by using GC at a current density of 400 mA cm^−2^ on Ni–N_4_/C, Ni–N_3_B_1_/C and Ni–N_4_–B/C. (c) Faradaic efficiencies for CH_4_ under various applied potentials. (d) *In situ* DEMS measurement for CH_4_ production. (e) Partial current densities for CH_4_ at different applied potentials. (f) *In situ* Raman spectra of Ni–N_3_B_1_/C at various potentials, recording the peak area change of HCO_3_^−^ and CO_3_^2−^. (g) pH values calculated from *in situ* Raman spectra, as a function of potential. (h) Long-term stability test for converting CO_2_ to CH_4_ at a current density of 300 mA cm^−2^ over Ni–N_3_B_1_/C. Inset shows the schematic illustration of the flow cell. The error bars in (a, c, e) are generated based on the mean ± standard deviation of three independent measurements.

We first focused on the CO_2_ electrolysis for Ni–NB/C with different catalyst loadings and varying amounts of boron. Data in Figs S18 and S19 show that the CO_2_RR selectivity is dependent on the current density. Adding 0.1 g of boric acid culminated in a peak CH_4_ Faradaic efficiency (FE) of 44.1 ± 2.3%, along with a catalyst loading of 1.0 mg cm^−2^, which was selected for subsequent electrochemical measurements. The linear sweep voltammetry (LSV) profiles exhibit significantly enhanced current densities in a CO_2_ atmosphere compared with N_2_, signifying the active involvement of CO_2_ molecules in the reaction (Fig. S20). We subsequently analysed the FEs of combined products on Ni–N_3_B_1_/C and its references (Fig. 3a and Figs S21–S26). For Ni–N_4_/C, CO is the major product, with negligible CH_4_, C_2_H_4_ and formate generation. In contrast, Ni–N_3_B_1_/C demonstrates distinctively different product selectivity of CO_2_RR, primarily producing CH_4_ across the whole investigated current densities, which could be demonstrated by a remarkable GC signal for CH_4_ (Fig. 3b). Moreover, this catalyst achieves >10% FE for C_2_ products, including ethanol, acetate and ethylene, within the potential range of −1.12 to −1.35 V vs. reversible hydrogen electrode (RHE) (Fig. S25). A plausible explanation is that the occurrence of C_2_ product formation in parallel with CH_4_ might originate from *CO–CHO generation through non-adsorbed CO coupling with *CHO [47]. Intriguingly, the Ni–N_4_–B/C catalyst manifests a product distribution that is analogous to Ni–N_4_/C, maintaining an excellent CO FE of ∼100% from 100 to 400 mA cm^−2^. It unmasks that B doping in the second shell performs a slight influence on the electronic structure of the Ni sites, which is insufficient to alter the reaction pathway that favors the CH_4_ production, as previously reported [48]. These observations disclose that the improved CH_4_ selectivity on Ni–N_3_B_1_/C, as opposed to Ni–N_4_/C and Ni–N_4_–B/C, presumably stems from the unique coordination environment of B in the first shell (Fig. 3c). Additionally, control experiments conducted with Ni–free BN/C highlight that the CO_2_RR product is dominated by H_2_ with marginal CO and CH_4_ generation, suggesting that the substrates are not responsible for CH_4_ production (Fig. S27). Concurrently, when the electroreduction measurement of Ni–N_3_B_1_/C was conducted in N_2_-saturated 1 M KOH electrolyte, only H_2_ signals were observed, thereby substantiating that the generation of CH_4_ was indeed derived from CO_2_ molecules (Fig. S28).

*In situ* differential electrochemical mass spectrometry (DEMS) was utilized to directly validate the CH_4_ formation. As illustrated in Fig. 3d and Fig. S29, Ni–N_3_B_1_/C performs lower overpotential and higher signal intensity for CO_2_-to-CH_4_ conversion relative to its counterparts, demonstrating that the first-shell B dopants indeed motivate the intrinsic activity for electrochemical CO_2_ reduction towards CH_4_. Data in Fig. 3e reveal that Ni–N_3_B_1_/C achieves a maximum partial current density of 242.3 mA cm^−2^ for CH_4_, whilst the value for Ni–N_4_–B/C is exclusively 58.9 mA cm^−2^ under the same conditions. In stark contrast, Ni–N_4_/C presents a negligible CH_4_ current density over the entire potential window. Moreover, the partial current densities of H_2_ and CO on these catalysts are also detailed in Fig. S30. Notably, under anion-exchange membrane conditions, the FE for CH_4_ further increased to 55.4 ± 0.5% with a partial current density of 332.4 mA cm^−2^ (Fig. S31), surpassing massive state-of-the-art CO_2_RR electrocatalysts (Fig. S32 and Table S2). Subsequently, we employed *in situ* Raman spectroscopy to monitor changes in the local pH during the reaction by analysing the peak area ratio of bicarbonate to carbonate. As depicted in Fig. 3f and g, and Fig. S33, a notable decrease in the local pH is indeed observed. This phenomenon can be well explained by the pH dependency of the hydrogenation process from *CO to *CHO species. A reduction in the pH increases the availability of protons, thereby lowering the activation energy barrier for *CHO production and eventually facilitating the formation of methane [49].

To acquire a high FE_CH4_ of >50%, we initially executed a long-term galvanostatic test at a current density of 500 mA cm^−2^ using the Ni–N_3_B_1_/C catalyst. Unfortunately, we noticed a significant decline in FE_CH4_ owing to the loss of hydrophobicity in the gas diffusion electrode (GDE), accompanied by rapid hydrogen evolution after only 1 h (Fig. S34). Afterward, we adjusted the applied current density to 300 mA cm^−2^ at the expense of the FE_CH4_ value. Remarkably, our Ni–N_3_B_1_/C catalyst exhibited superior stability, as evidenced by the absence of noticeable fluctuations in both CH_4_ FE and overpotential over 34 operating hours (Fig. 3h). A suite of structural and morphological analyses also revealed that, following electrolysis, Ni–N_3_B_1_/C still retained its monatomic dispersion (Fig. S35).

### Mechanism investigation

**Figure 4.**
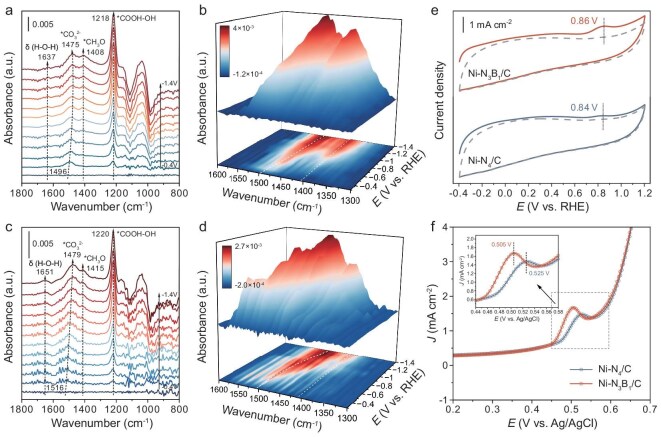
Investigation of key intermediates. *In situ* ATR-SEIRAS spectra recorded at various applied potentials for (a, b) Ni–N_3_B_1_/C and (c, d) Ni–N_4_/C in CO_2_-saturated 0.1 M KHCO_3_ electrolyte. (e) Electrochemical CO-stripping curves for Ni–N_3_B_1_/C and Ni–N_4_/C in 0.1 M KHCO_3_ electrolyte. The solid and dashed curves correspond to the first (presence of CO adsorbed on the catalyst) and second (absence of CO absorbed on the catalyst) scans, respectively. (f) Single oxidative LSV scans in N_2_-saturated 0.1 M KOH solution for Ni–N_3_B_1_/C and Ni–N_4_/C.

To better understand the catalytic mechanism of the promotive effect of B doping, *in situ* attenuated total reflection surface-enhanced infrared absorption spectroscopy (ATR-SEIRAS) was employed to monitor the reactive species and key intermediates over the catalyst surface. As depicted in Fig. 4a, for the Ni–N_3_B_1_/C catalyst, the peak around ∼1637 cm^−1^ corresponds to bending vibrations of the interfacial H_2_O, suggesting the participation of water in the electrolysis process [18,50]. With the decrease in applied potentials, a gradually enhanced band at ∼1218 cm^−1^ can be ascribed to the OH deformation of *COOH, which is generally accepted as the pivotal intermediate for CO_2_RR to CO and CH_4_ [51,52]. Furthermore, the peak centered at ∼1475 cm^−1^, with an obvious Stark effect, is possibly regarded as the CO_3_^2−^ species [53,54]. Similar species are also detected in Ni–N_4_/C, but with lower characteristic intensities (Fig. 4c). Significantly, however, several new peaks associated with *CH_3_O (∼1408 cm^−1^) [55–57] and CH*_x_* (∼2866 and ∼2953 cm^−1^) [58], which are the crucial intermediates for methane formation, are distinctly observed only at −0.5 V vs. RHE over Ni–N_3_B_1_/C (Fig. 4b and Fig. S36a). Conversely, for the Ni–N_4_/C catalyst, a weak *CH_3_O signal is not shown until −1.0 V vs. RHE, with no significant feature of CH*_x_* across the entire potential region (Fig. 4d and Fig. S36b). This phenomenon corresponds well with our experimental results, indicating that the introduction of B contributes to the formation of hydrogenated intermediates, thereby steering different product distributions.

To examine the influence of this modification on CO-adsorption capability, we conducted electrochemical CO-stripping experiments (Fig. 4e) [59,60]. The Ni–N_3_B_1_/C catalyst exhibits a more pronounced and more positive CO-stripping peak than the Ni–N_4_/C catalyst, indicating a stronger CO-binding ability, which is advantageous for the subsequent hydrogenation of CO to generate methane. On the other hand, we performed OH^−^-adsorption measurements to further evaluate the binding affinity of *CO_2_^−^ on catalysts. As shown in Fig. 4f, Ni–N_3_B_1_/C exhibits a more positive potential for surface OH^−^ adsorption compared with Ni–N_4_/C, implying a more effective stabilization of *CO_2_^−^ on Ni–N_3_B_1_/C. Altogether, these findings prove that Ni–N_3_B_1_/C is capable of boosting CO_2_ molecule activation and enhancing CO adsorption, ultimately accelerating the conversion of CO_2_ to CH_4_.

**Figure 5.**
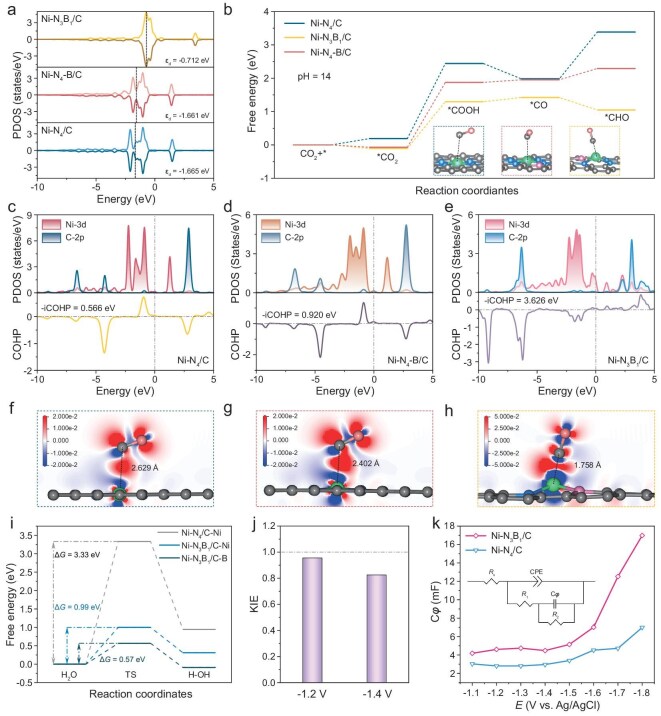
DFT calculations. (a) Projected density of states (PDOS) and (b) Gibbs free energy profiles of CO_2_RR to CH_4_ pathways on Ni–N_4_/C, Ni–N_4_–B/C and Ni–N_3_B_1_/C. The d-band centers were calculated as −0.712 eV for Ni–N_3_B_1_/C, −1.661 eV for Ni–N_4_–B/C and −1.665 eV for Ni–N_4_/C. PDOS for Ni 3*d* orbital and 2*p* orbital of adsorbed CO as well as crystal orbital Hamilton population (COHP) analysis of the Ni–C bond for (c) Ni–N_4_/C, (d) Ni–N_4_–B/C and (e) Ni–N_3_B_1_/C. Two-dimensional differential charge density cross section of (f) Ni–N_4_/C, (g) Ni–N_4_–B/C and (h) Ni–N_3_B_1_/C. (i) Energy barrier profiles for H_2_O dissociation process on Ni–N_4_/C and Ni–N_3_B_1_/C. (j) KIE of CO_2_RR to CH_4_ on Ni–N_3_B_1_/C. (k) Calculated *C*_φ_ of Ni–N_4_/C and Ni–N_3_B_1_/C at various potentials.

Previous investigations into Ni SACs have predominantly resulted in CO formation, while the subsequent hydrogenation to methane remains challenging. This is largely attributed to the relatively weak adsorption energy of *CO intermediates on Ni sites. To enhance methane production, it is imperative to improve the adsorption of *CO intermediates to enable further hydrogenation to *CHO. This process heavily relies on optimizing the electronic structure of the isolated Ni sites. Utilizing DFT calculations, we explored the influence of the coordination environment around the Ni center on the catalytic mechanism for CO_2_ electroreduction to CH_4_. Based on the EXAFS fitting results, three models, namely Ni–N_4_–C, Ni–N_4_–B–C and Ni–N_3_B_1_–C, were constructed to represent the Ni–N_4_/C, Ni–N_4_–B/C and Ni–N_3_B_1_/C catalysts, respectively. All computational structural models are illustrated in Figs S37–S39. The projected density of states (PDOS) analysis (Fig. 5a) indicates that the incorporation of B into the first shell significantly elevates the d-band center of Ni atoms, resulting in electron delocalization, which enhances hybridization between the d orbital and the anti-bonding 2π* orbital of CO (known as d→2π* backdonation), facilitating more profound CO_2_ reduction [61–63]. Subsequently, we adopted a computational hydrogen electrode model to investigate the free energy diagram for the conversion of CO_2_ to *CHO—a crucial intermediate for CH_4_ production (Fig. 5b). The rate-determining step for all these catalysts was identified as the conversion from *CO_2_ to *COOH and this step was favorable on Ni–N_3_B_1_/C. After *CO formation, *CO will hydrogenate to *CHO, which is exothermic at the Ni–N_3_B_1_/C site, while endothermic at the Ni–N_4_/C and Ni–N_4_–B/C sites, indicating a preference for CH_4_ production instead of CO on the Ni–N_3_B_1_/C catalyst. This observation is attributed to the enhanced *CO-adsorption energy (Fig. S40). Moreover, the free energies for each intermediate on Ni–N_3_B_1_/C were found to be lower than those on Ni–N_4_/C and Ni–N_4_–B/C, further emphasizing the superiority of B coordination in the first shell for promoting CO_2_ activation and intermediate stability.

Given the pivotal role of *CO adsorption in regulating product selectivity, we further employed PDOS and crystal orbital Hamilton population (COHP) analyses to elucidate the interactions between *CO and catalysts. Data in Fig. 5c–e illustrate an enhanced orbital overlap between Ni 3*d* for Ni–N_3_B_1_/C and C 2*p* for the adsorbed *CO compared with Ni–N_4_/C and Ni–N_4_–B/C. COHP analysis reveals a substantial anti-bonding contribution near the Fermi energy level for Ni–N_4_/C and Ni–N_4_–B/C whereas Ni–N_3_B_1_/C shows minimal anti-bonding contributions. Further integration of the COHP confirms a more robust Ni–C bond interaction on Ni–N_3_B_1_/C, which is advantageous for *CO adsorption and subsequent hydrogenation to CH_4_. This conclusion is further supported by visualized differential charge distribution (Fig. S41). A more pronounced electron transfer and shorter Ni–C bond length (Table S3) between the Ni center and *CO intermediates are observed in the presence of the first-shell B atom, suggesting that the introduction of B can improve d→2π* backdonation and thus stabilize the *CO species (Fig. 5f–h). To quantify electron transfer during CO adsorption, we conducted a Mulliken charge population analysis. Intriguingly, B also functioned as an additional electron donor for *CO adsorption, alongside Ni sites (Table S4), suggesting that the adsorption of intermediates resulted from the synergistic effect of the Ni and B sites.

For the formation of CH_4_, enhancing CO adsorption is crucial, while the availability of protons is equally essential. Under alkaline conditions, water molecules serve as the sole proton source. Improving water dissociation can supply more available protons for methane production. In this study, we selected the free energy change (Δ*G*) for H_2_O dissociation as a crucial descriptor to assess the capability of proton supply in the reaction. As shown in Fig. 5i, the Δ*G* for H_2_O dissociation at both the Ni (0.99 eV) and B (0.57 eV) sites on Ni–N_3_B_1_/C was significantly lower than that on Ni–N_4_/C (3.33 eV), demonstrating that B doping accelerates water dissociation and improves the proton-feeding ability, facilitating the generation of key intermediates such as *COOH and *CHO. This finding aligns with prior research indicating that the Lewis acid B sites, owing to their oxygenophilic properties, favor the adsorption of water molecules and the cleavage of H–OH bonds [26,64]. Subsequently, the pivotal role of B atoms in water activation and protonation processes was further corroborated through kinetic isotope effect (KIE) measurements of H/D on Ni–N_3_B_1_/C (Fig. 5j and Fig. S42). The measured KIE values were <1.0 at both −1.2 V and −1.4 V vs. RHE, indicating that hydrogenation is not the rate-determining step on the Ni–N_3_B_1_/C catalyst [51,65,66]. In addition, we carried out *in situ* electrochemical impedance spectroscopy (EIS) to examine the availability of adsorped hydrogen (H*) on the catalyst surface. A double-parallel equivalent circuit model was utilized to simulate the Nyquist plots and adsorption pseudocapacitance (*C*_φ_) was applied to quantify the H* availability (Fig. S43) [67,68]. Compared with Ni–N_4_/C, Ni–N_3_B_1_/C exhibited greater H* availability, which could provide sufficient H* for the hydrogenation process (Fig. 5k). These comprehensive analyses collectively indicate that the generation of CH_4_ on Ni–N_3_B_1_/C benefits from both the enhanced *CO adsorption and accelerated water-dissociation kinetics.

## CONCLUSION

In conclusion, we developed and synthesized a Ni single-atom catalyst coordinated with boron and nitrogen in the first shell. Notably, the presence of boron in the first shell facilitated the generation of CH_4_ whereas its absence or presence in the secondary shell led exclusively to CO formation. This observation underscores the crucial role of the coordination environment at the Ni sites in steering the reaction pathway. Combined with *in situ* characterization and DFT calculations, it was demonstrated that the introduction of B into the first shell significantly elevated the d-band center of Ni, induced its electron delocalization and enhanced CO adsorption. Furthermore, boron served as an active site for water dissociation, thereby accelerating the kinetics of proton-coupled electron transfer. Taken together, the Ni–N_3_B_1_/C catalyst successfully achieved > 50% CH_4_ FE on isolated Ni sites for the first time. This study highlights the significant impact of local chemical microenvironment regulation on reaction pathways, while also providing profound insights into the design of non-copper-based electrocatalysts for promoting the generation of hydrocarbons.

## Supplementary Material

nwaf173_Supplemental_File
