# Supplementary material for: Modulating the local microenvironment over isolated nickel sites through first-shell coordination to regulate the reaction pathway of CO2 electroreduction
Source: Natl Sci Rev. 2025 May 6;12(7):nwaf173. doi: 10.1093/nsr/nwaf173 (PMC12202009; doi:10.1093/nsr/nwaf173)
Supplement: nwaf173_Supplemental_File [file nwaf173_supplemental_file.pdf]

Supporting Information for

Modulating the Local Microenvironment over Isolated Nickel Sites through First-Shell Coordination to Regulate the Reaction Pathway of CO_2 Electroreduction

Yan Kong[1,2], Xinmei Jia[1,2], Xiaoyan Chai[1], Zhi Chen[1], Chunyan Shang[1], Xingxing Jiang[1], Huizhu Cai[1], Lingyan Jing[1], Qi Hu[1], Hengpan Yang[1], Xue Zhang[1], Chuanxin He[1,]*

[1]College of Chemistry and Environmental Engineering, Shenzhen University, Shenzhen, Guangdong, 518060, China

[2]Department of Chemical Physics, University of Science and Technology of China, Hefei, Anhui, 23002, China

*Correspondence should be addressed to: email: hecx@szu.edu.cn

Experimental Procedures

1. Chemicals and materials

All reagents were directly used as received without any further purification in our entire experiments. Nickel acetylacetonate (Ni(acac)$_2$, 95%), urea (CH_4ON_2, AR), boric acid (H_3BO_3, ≥99.8%), nickel acetate tetrahydrate ($NiC_4H_6O_4 \cdot 4H_2O$, 99%), deuterium oxide (D_2O, 99.9%), sodium deuteroxide (NaOD) and potassium hydroxide (KOH, 95%) were purchased from Shanghai Aladdin Biochemical Technology Co., Ltd. Pluronic F127 and 1, 10-phenanthroline ($C_{12}H_8N_2$, 99.98%) were obtained from Sigma-Aldric. Carbon black was purchased from Shanghai Macklin Biochemical Co., Ltd. Ethanol (C_2H_5OH, ≥99.7%) was received from Guangzhou Guanghua Sci-Tech Co., Ltd. High-purity CO_2 and N_2 gases were provided by Shenzhen Huatepeng Special Gases Co., Ltd. The ultrapure water with a resistivity of 18.25 MΩ cm^{-1} was used in all experiments.

2. Synthesis of catalysts

2.1 Synthesis of Ni-N$_3$B$_1$/C

In a typical synthesis process [1], 3 g of urea and 0.1 g of H_3BO_3 were dispersed in a 10 mL mixture containing equal volumes of ultrapure water and ethanol. Whereafter, 0.2 g of Pluronic F127 and 6.9 mg of Ni(acac)$_2$ were added into the preceding solution under mechanical agitation. The homogeneous mixture was heated at 80 ℃ to evaporate solvent. Following this, the obtained powder (1 g) was combined with 180 mg of carbon black and re-dispersed in a 40 mL ethanol-water solution. Similarly, the dispersion was heated under 70 ℃ with continuous stirring until a black powder was formed. The collected product was thoroughly ground in an agate mortar and transferred to a corundum crucible. Finally, the precursor was pyrolyzed in N_2 protection at 800 ℃ for 2 h. After cooling to room temperature naturally, the final product was collected and denoted as Ni-N$_3$B$_1$/C.

For the preparation of samples with different amounts of boric acid, the synthesis conditions were kept consistent except for the altered quantities of H_3BO_3, which were 0.05 g and 0.5 g for Ni-B$_{0.05}$N/C and Ni-B$_{0.5}$N/C, respectively.

2.2 Synthesis of Ni-N$_4$/C

The Ni-N$_4$/C catalyst was fabricated via a similar procedure as that of Ni-N$_3$B$_1$/C except without the addition of boric acid.

2.3 Synthesis of Ni-N$_4$-B/C

The $Ni-N_4-B/C$ catalyst was prepared undergoing a different precursor process. 21.25 mg of $Ni(OAc)_2 \cdot 4H_2O$ and 51 mg of 1,10-phenantroline were dissolved in 5 mL ethanol. The solution was stirred for 30 min to form a Ni(phen) complex. Then the Ni(phen) complex solution was added into the uniform mixture including H_3BO_3, urea, and F127 under magnetic stirring. Subsequently, the remaining operation aligned to that of $Ni-N_3B_1/C$.

3. Physicochemical characterization

The structure of as-prepared catalysts was characterized via powder X-ray diffraction (PXRD) on a PANalytical diffractometer with Cu-Kα (λ = 0.154178 Å). The surface morphologies were examined by field emission scanning electron microscopy (SEM, JEOL-7800F) and transmission electron microscopy (TEM, JEM-2100F). Double spherical aberration-corrected high-angle annular dark-field scanning transmission electron microscopy (HAADF-STEM, Titan Cubed Themis G2 300) was further performed to obtain elaborate atomical morphology. The operated accelerating voltage was 200 kV. *Ex situ* Raman spectra were taken on the microscopic confocal Raman spectrometer of Jobin Yvon Horiba HR800 with an excitation wavelength of 532 nm. Element analysis of Ni in all electrocatalysts was detected by inductively coupled plasma optical emission spectroscopy (ICP-OES) with an Agilent 5110 spectrometer. The N_2 adsorption-desorption isotherms were carried out on the BelSorp Max apparatus to analyze the specific surface area of materials. The surface chemical states were determined via X-ray photoelectron spectroscopy (XPS, Thermo Fisher Scientific) with Al Kα = 1486.6 eV as the exciting source. The C 1*s* peak of 284.8 eV was used to calibrate all binding energies. The X-ray absorption spectra (XAS) containing X-ray absorption near-edge structure (XANES) and extended X-ray absorption fine structure (EXAFS) at Ni K-edge were collected at the beamline BL14W1 station of the Shanghai Synchrotron Radiation Facility.

4. Preparation of cathode electrodes

To prepare an electrocatalyst applied in a flow cell device, we deposited 10 mg of Ni-based sample mixed with 80 μL of 5 wt% Nafion solution in 1 mL of ethanol under ultrasonic dispersion for 90 min, yielding a homogeneous slurry. Then we painted the obtained slurry on the commercial gas diffusion layer (GDL) using an airbrush. After drying, the catalyst loading was determined by weighing the mass of carbon paper before and after the spraying, ~1 mg cm^{-2}.

5. Electrochemical measurements

All electrochemical measurements were carried out in a 1 cm^2 flow cell configured with

a three-electrode system. In this setup, a proton exchange membrane (Nafion 117) was used to separate the anodic and cathodic chambers, and in certain instances, an anion exchange membrane (Fumasep FAA-3-PK-130) was also employed. An IrO_x/Ti mesh and an Ag/AgCl electrode were utilized as the counter electrode and reference electrode, respectively. Unless otherwise stated, all applied potentials were calibrated with respect to the RHE through the Nernst equation: E (vs. RHE) = E (vs. Ag/AgCl) + 0.197 + 0.0591 × pH. Meanwhile, 70% solution resistance taken by electrochemical impedance spectroscopy was compensated in all trials. 1 M KOH was used as the electrolyte and circulated through both the anodic and cathodic chambers by a dual-channel peristaltic pump. High-purity CO_2 gas was supplied to the cathodic chamber with a constant flow rate of 20 mL min^{-1} monitored via a mass flow controller.

The gaseous products were determined by a gas chromatograph (GC-2014, Shimadzu) integrated with a flame ionization detector (FID) and a thermal conductivity detector (TCD). The liquid products were analyzed by ^1H NMR (Bruker AVANCE III 500 MHz) with a water suppression program, where 400 μL electrolyte was mixed with 100 μL D_2O and 100 μL DMSO solution.

OH^- adsorption was measured using a single electrolysis cell configured with a three-electrode system. Specifically, A platinum sheet served as the counter electrode and an Ag/AgCl electrode as the reference electrode. The working electrodes were prepared by depositing the Ni-N_4/C and Ni-N_3B_1/C samples onto a 1 × 1 cm^2 piece of commercial carbon paper. During the measurements, OH^- adsorption on the catalyst surface was monitored by linear sweep voltammetry in a N_2-saturated 0.1 M KOH electrolyte. The potential was scanned from 0.2 V to 0.7 V vs. Ag/AgCl at a scan rate of 10 mV s^{-1}.

6. In situ Raman measurements

In situ Raman spectra were performed on a Renishaw confocal Raman spectrometer with a 633 nm laser for excitation. The laser power was set at 25 mW, and the Raman scattering was collected by the steady-state mode. To enhance the signal-to-noise ratio, every spectrum was the result of 50 accumulations, with each acquisition lasting 1 second. During the measurement, a homemade flow cell with a quartz window was employed, where Pt wire and Ag/AgCl served as the counter and reference electrode, respectively. 1 M KOH electrolyte saturated by high-purity CO_2 was circulated into the setup by a peristaltic pump, and CO_2 was continuously bubbled into the KOH solution. Finally, the Raman spectra were recorded after stabilization of ~5 min following electrolysis at each applied potential.

7. In situ DEMS measurements

In situ differential electrochemical mass spectroscopy experiments were conducted to continuously monitor CO_2 and its reaction products during real-time electrochemical reactions. The typical electrochemical cell was equipped with three electrodes. The reference electrode and counter electrode were Ag/AgCl and Pt wire, respectively. The working electrode was attained by drop-casting the catalyst ink onto the gold-plated membrane. Before the electrochemical test, meticulous leak detection for this setup with electrolyte added was warranted. Subsequently, high-purity CO_2 was introduced into 1 M KOH solution for at least 30 min, and CO_2 gas was also consecutively bubbled in the electrolyte during the experiment. The linear sweep voltammetry was executed to measure the fragment ion signals with a sweeping rate of 5 mV s^{-1} from 0 to −1.8 V vs. RHE.

8. In situ ATR-SEIRAS measurements

The *in-situ* ATR-SEIRAS experiment was conducted using a Nicolet iS50 (Thermo Fisher Scientific) spectrometer equipped with a liquid nitrogen-cooled MCT detector. A gold film of ~60 nm thickness was chemically deposited on a silicon prism to prepare the substrate. The deposition process began with the silicon prism being immersed in piranha solution (a 7:3 volumetric ratio of 98% H_2SO_4 and 30% H_2O_2) for 2 h, followed by polishing with 0.05 μm Al_2O_3 powder to achieve a hydrophobic surface. After that, the silicon prisms were thoroughly rinsed with deionized water to remove any residual Al_2O_3 particles. Subsequently, the silicon prism was dried and soaked for 90 s in an NH_4F solution. The treated prism was then immersed in an aqueous solution including $HAuCl_4$ and 2 wt% HF under a 55 ℃ water bath for 5 min to allow for chemical deposition of the gold film. After deposition, the gold-coated silicon prism was washed with deionized water and naturally dried. For the preparation of the catalyst ink, 10 mg of either Ni-N_4/C or Ni-N_3B_1/C powder was dispersed in 1 mL of ethanol, followed by the addition of 30 μL of Nafion solution. The mixture was sonicated for 1 h to form a uniform suspension, after which 400 μL of the ink was drop-cast onto the Au film. The obtained electrode was assembled into a custom-made spectroelectrochemical cell as the working electrode, with a Pt mesh as the counter electrode and a saturated Ag/AgCl as the reference electrode. Before the *in-situ* ATR-SEIRAS measurements, the working electrode was activated in an N_2-saturated 0.1 M $KHCO_3$ by CV cycles. The spectra were recorded at different applied potentials from −0.4 to −1.4 V vs. RHE with a resolution of 4 cm^{-1}. The spectra under open circuit potential (OCP) were used as the baseline correction.

9. DFT Calculations

DFT calculations were conducted using the first-principles CASTEP packages [2]. For

all calculations, the generalized gradient approximation (GGA) [3] with the Perdew-Burke-Ernzerhof (PBE) formula [4] was used to describe the exchange-correlation potential, augmented with the DFT-D correction for dispersion interactions. This work included spin-polarized calculations. The cutoff energy for atomic wave functions was set at 450 eV and ultrasoft pseudopotentials were selected to ensure the accuracy of the geometry optimizations. Meanwhile, the Broyden-Fletcher-Goldfarb-Shanno (BFGS) algorithm was employed to search for the ground state of supercells [5]. The convergence criteria for all geometry optimizations required that the Hellmann-Feynman forces on atoms should not exceed 0.02 eV $Å^{-1}$, and the total energy difference and inter-ionic displacement should be less than 10^{-5} eV $atom^{-1}$ and 0.001 Å, respectively.

The computational hydrogen electrode (CHE) method, developed by Nørskov [6], was used to calculate the free energy of each elementary step. This method offers an efficient approach to circumvent the explicit treatment of solvated protons. In this method, zero voltage is defined based on the reversible hydrogen electrode (RHE) reaction:

$$H^+ + e^- \leftrightarrow \frac{1}{2}H_2$$

All the calculated free energies of the reaction steps were obtained using the following equation:

$$\Delta G = \Delta E + \Delta ZPE - T\Delta S$$

where ΔE represents the total energy calculated from DFT calculations, ΔZPE is the change of zero-point energy, and ΔS is the change in entropy. In this study, all calculations were performed at a potential of U = 0 V and a pH of 14, to elucidate the intrinsic electroactivity differences in CO_2RR.

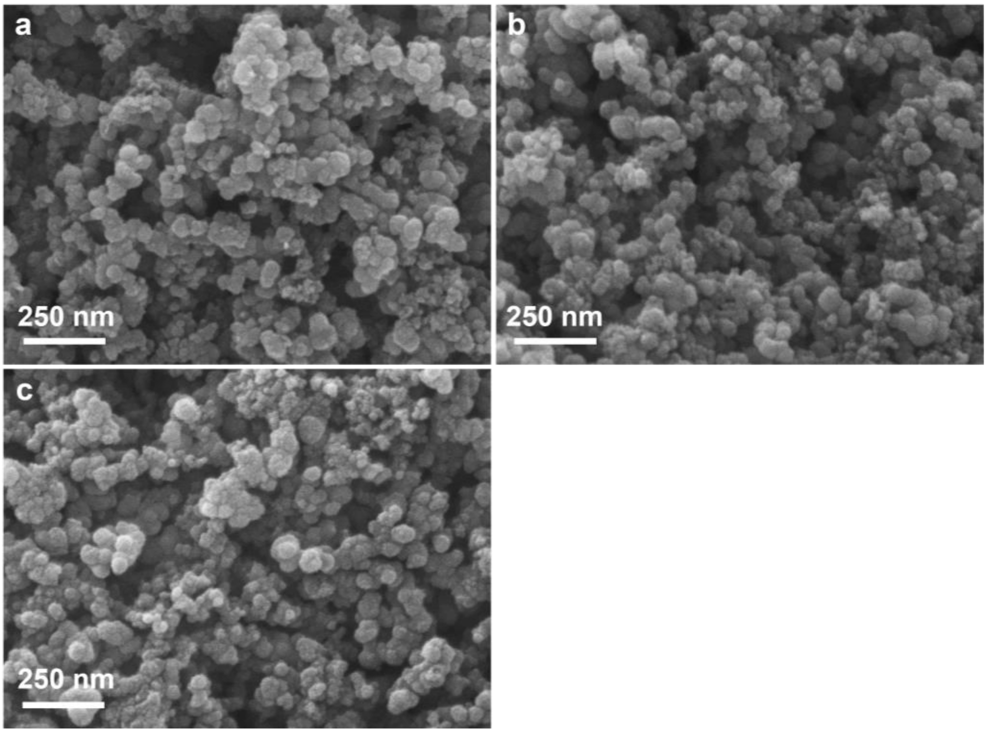

Figure S1. SEM images of Ni-N_4/C (a), Ni-N_3B_1/C (b), and Ni-N_4-B/C (c).

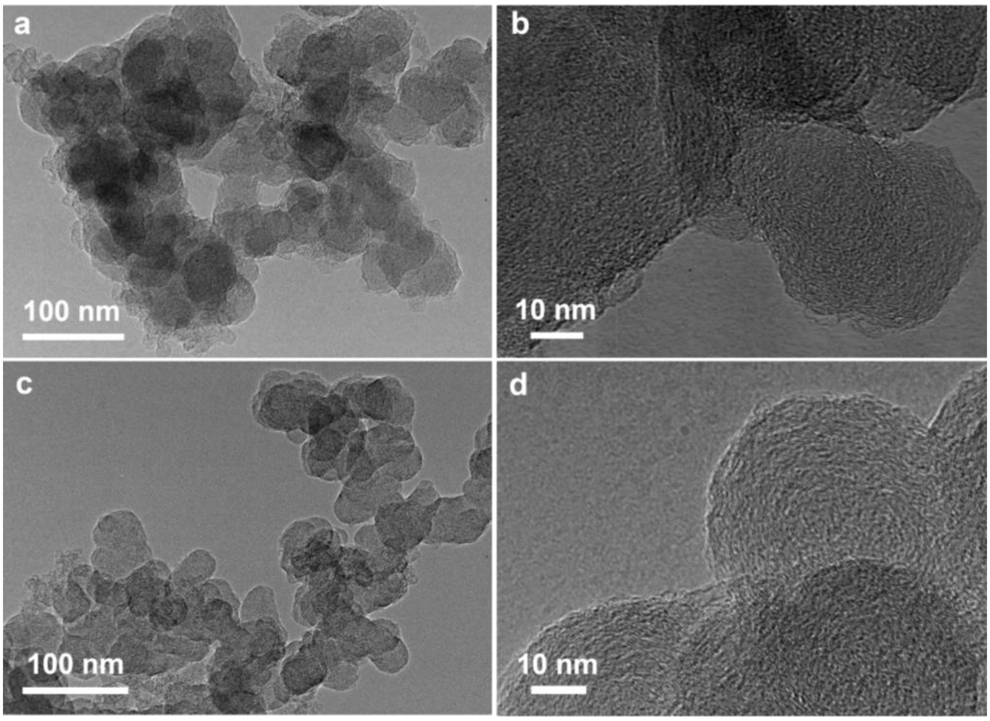

Figure S2. Low-magnification TEM images of Ni-N_4/C (a) and Ni-N_4-B/C (c). High-magnification images of Ni-N_4/C (b) and Ni-N_4-B/C (d).

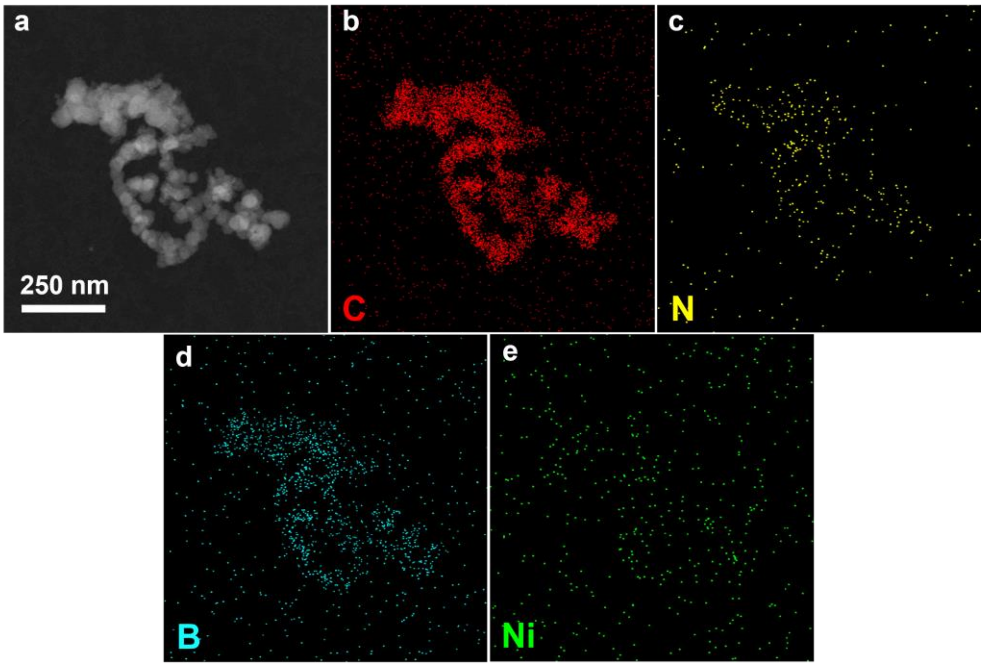

Figure S3. STEM image and the corresponding EDS mappings of Ni-N_3B_1/C.

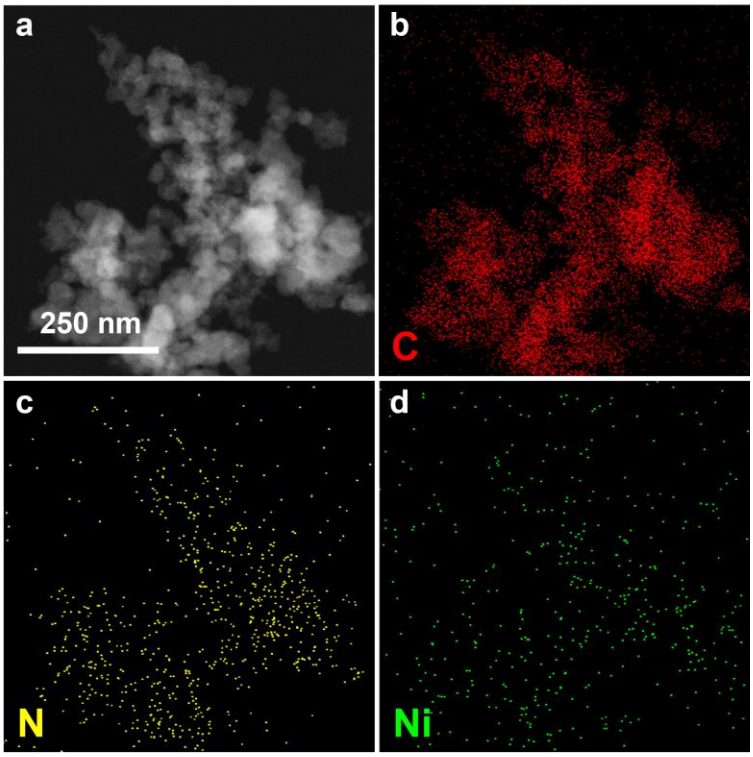

Figure S4. STEM image and the corresponding EDS mappings of Ni-N_4/C.

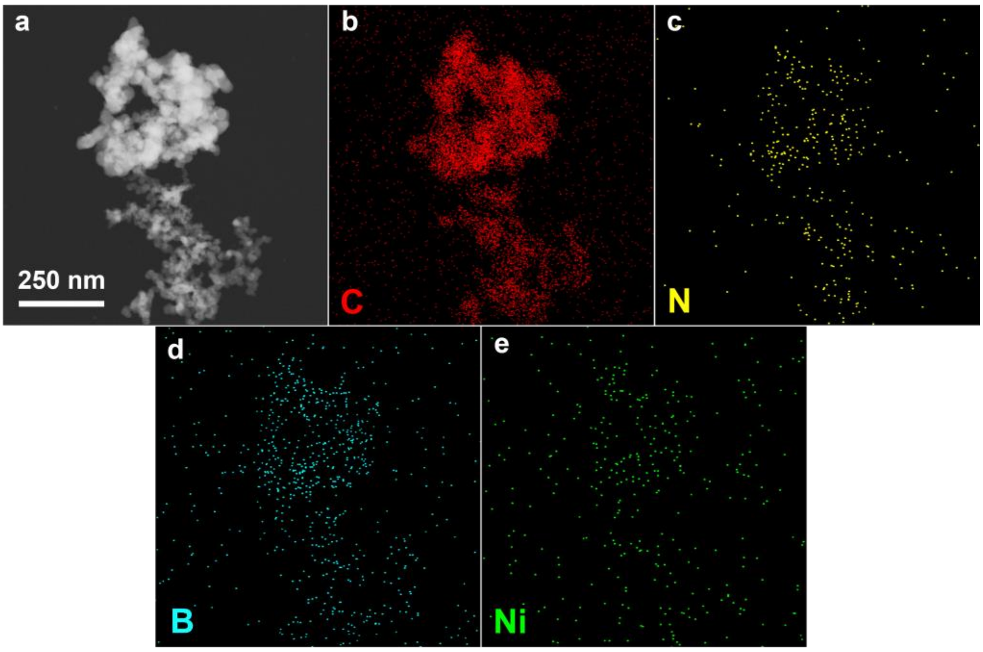

Figure S5. STEM image and the corresponding EDS mappings of Ni-N_4-B/C.

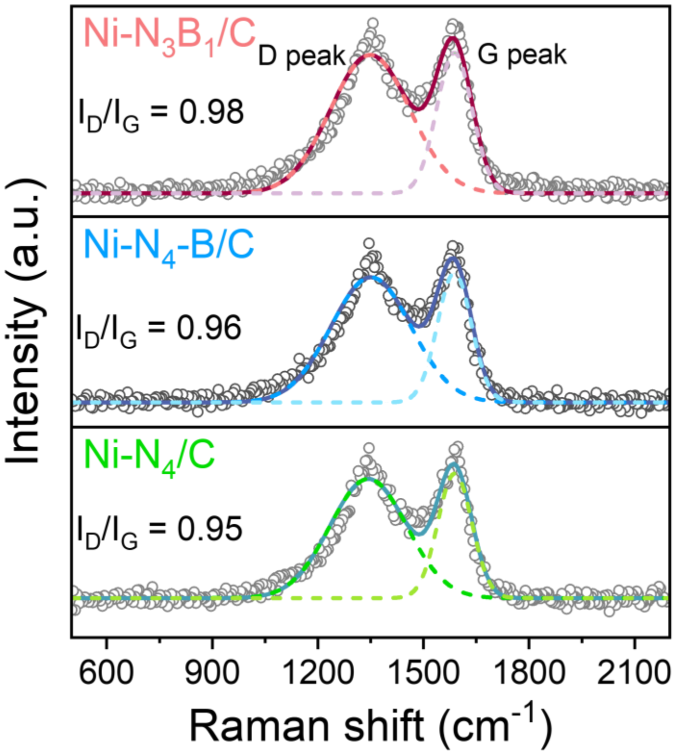

Figure S6. The Raman spectra of Ni-N_4/C, Ni-N_4-B/C, and Ni-N_3B_1/C. The I_D/I_G is used to denote the intensity ratio of the D peak to the G peak.

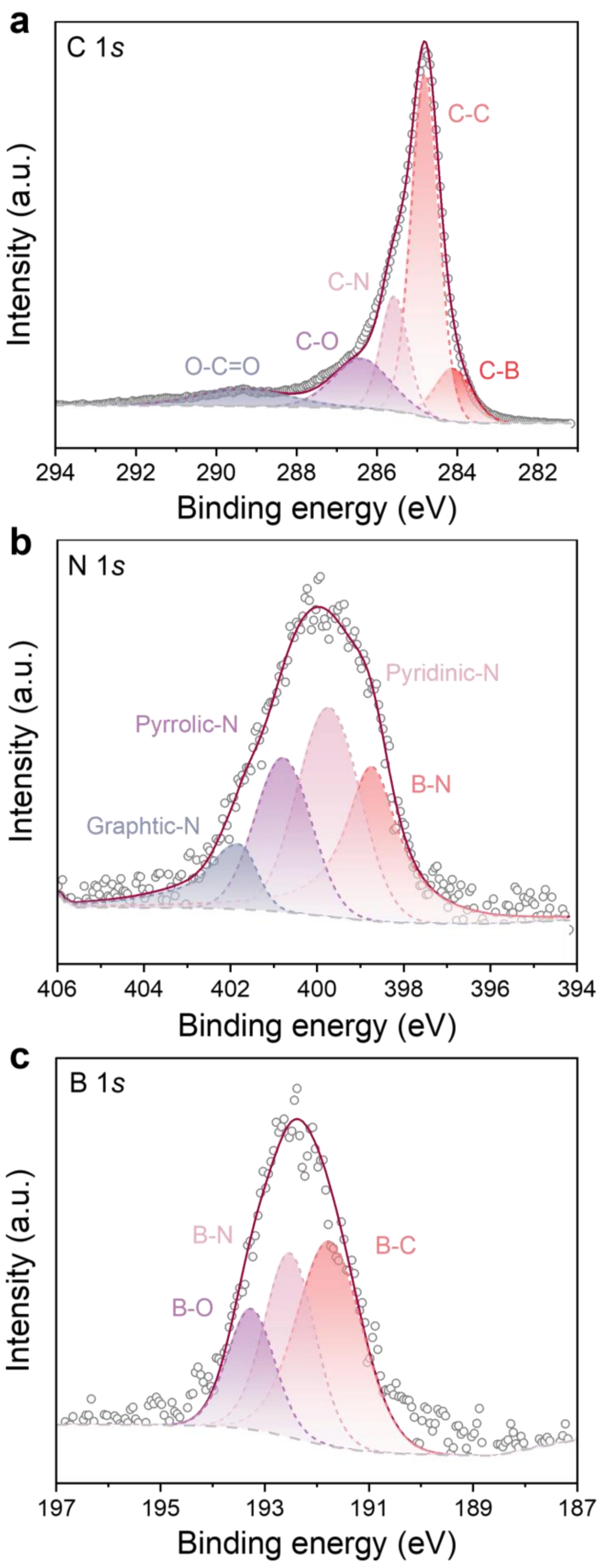

Figure S7. High-resolution C 1*s* (a), N 1*s* (b), and B 1*s* (c) spectra for Ni-N_3B_1/C.

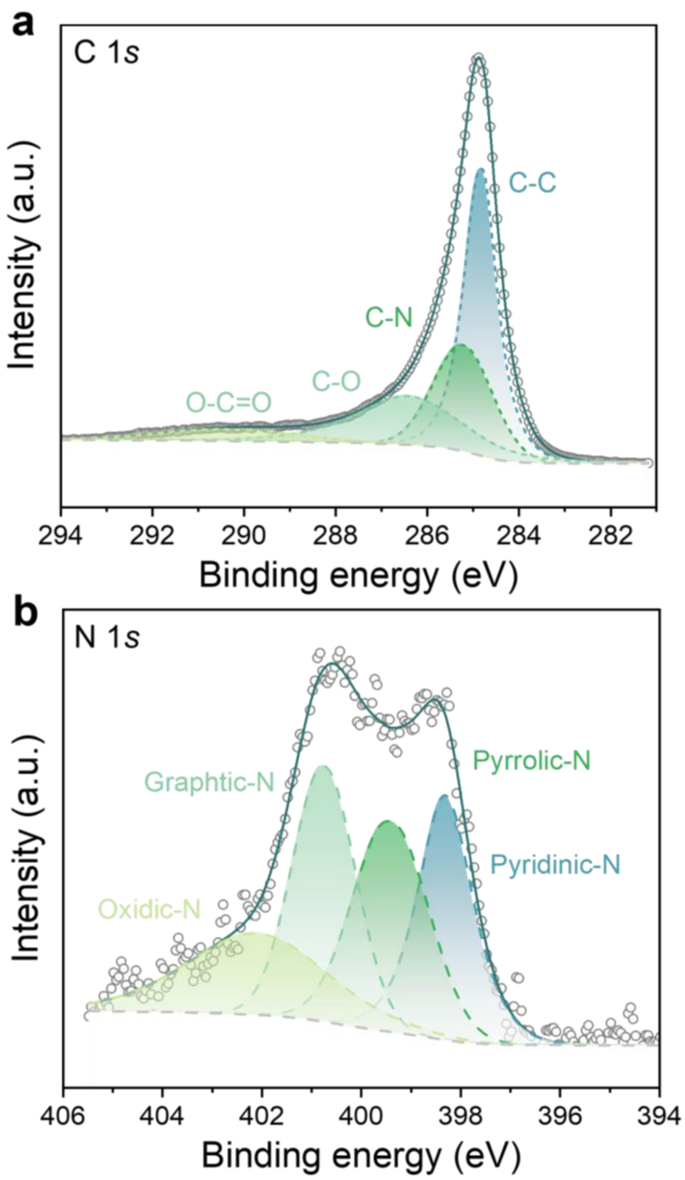

Figure S8. High-resolution C 1*s* (a) and N 1*s* (b) spectra for Ni-N_4/C.

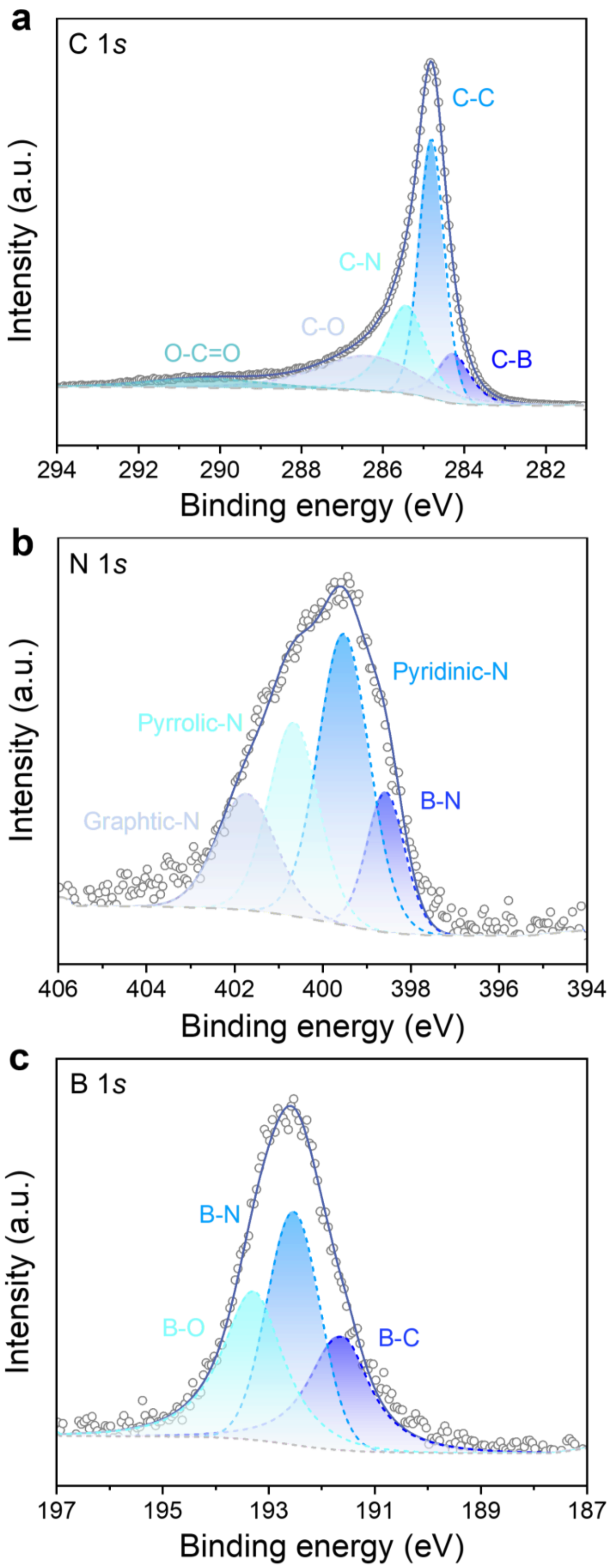

Figure S9. High-resolution C 1*s* (a), N 1*s* (b), and B 1*s* (c) spectra for Ni-N_4-B/C.

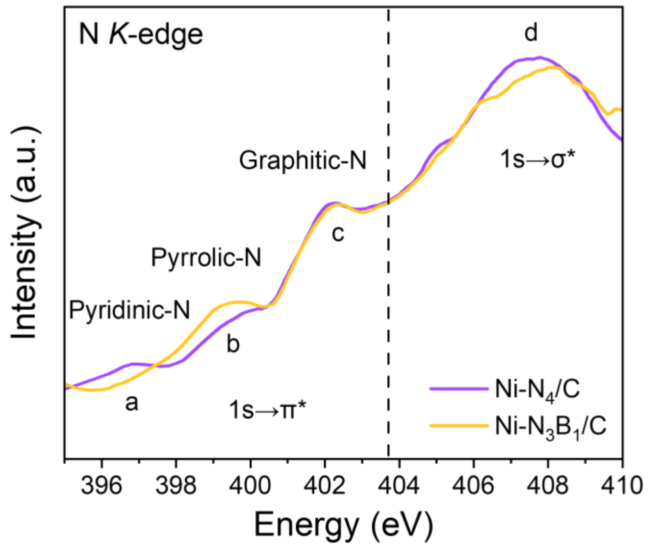

Figure S10. The N *K*-edge XANES spectra of Ni-N_4/C and Ni-N_3B_1/C catalysts.

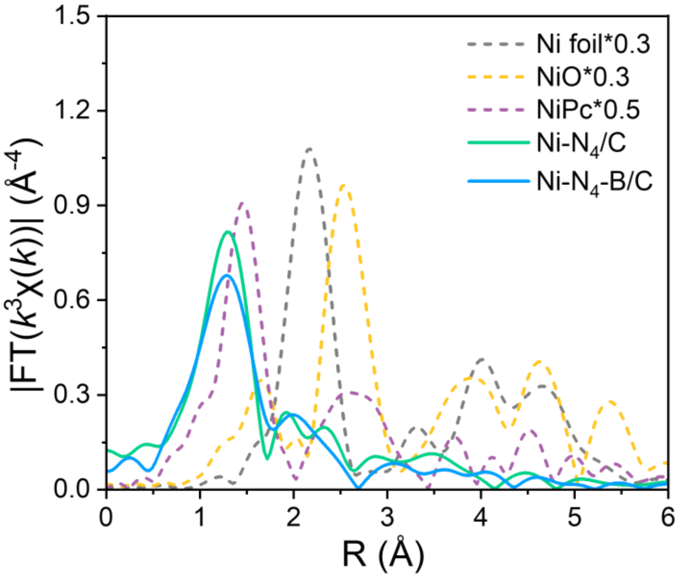

Figure S11. Fourier transformed EXAFS spectra of Ni-N_4/C and Ni-N_4-B/C.

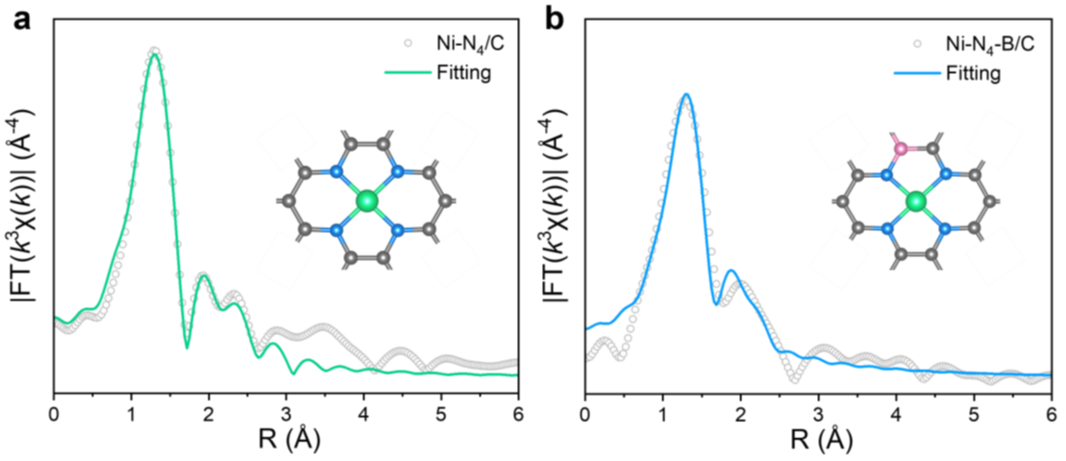

Figure S12. The EXAFS fitting curves in *R* space of Ni-N_4/C (a) and Ni-N_4-B/C (b).

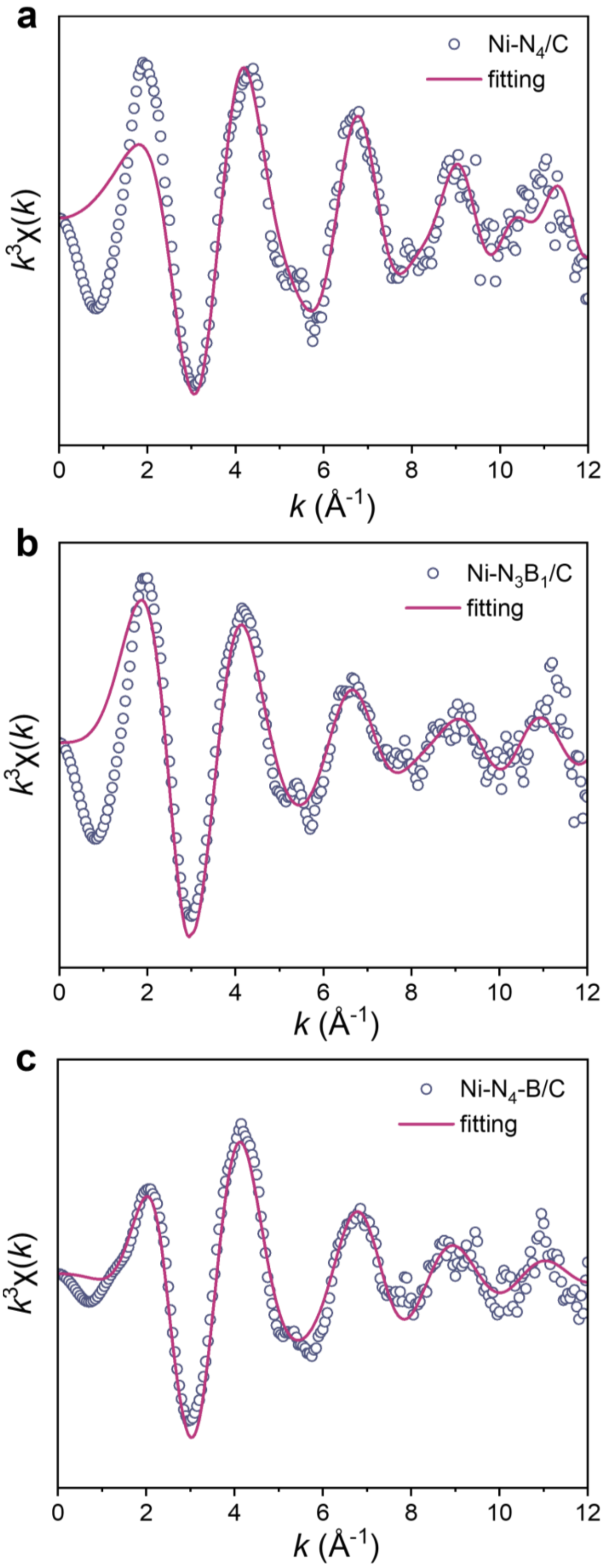

Figure S13. The EXAFS fitting curves in *k* space of Ni-N_4/C (a), Ni-N_3B_1/C (b), and Ni-N_4-B/C (c) at the Ni *K*-edge.

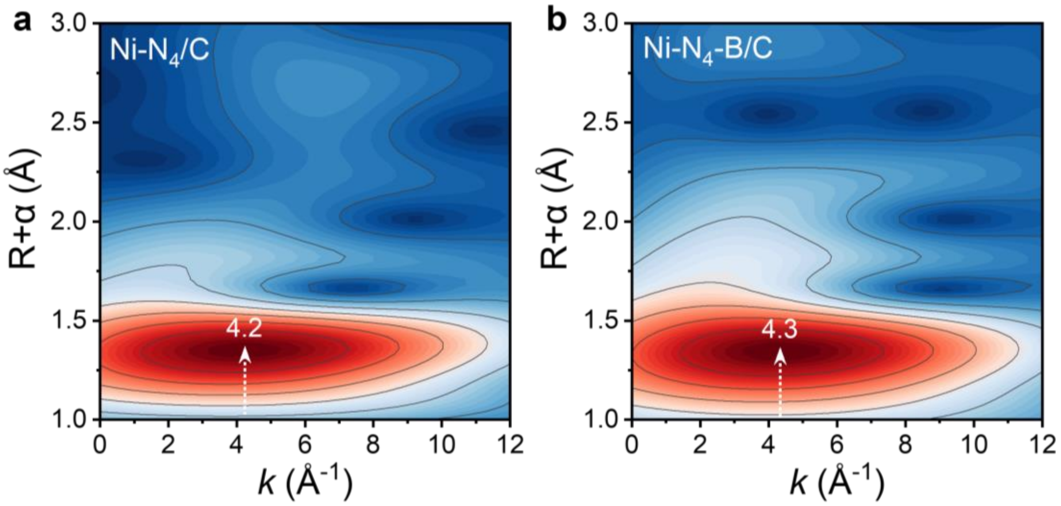

Figure S14. Wavelet transform EXAFS plots of Ni-N_4/C (a) and Ni-N_4-B/C (b).

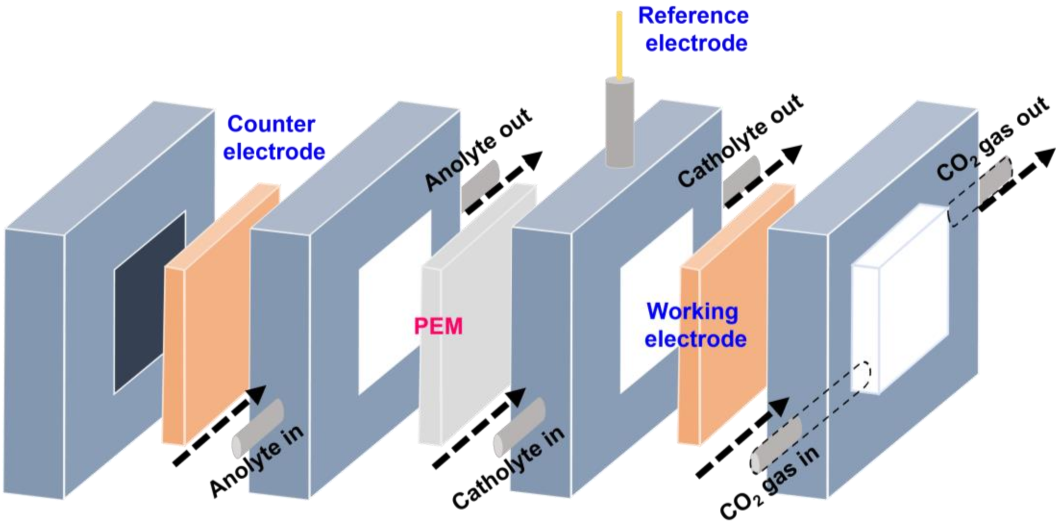

Figure S15. Schematic diagram of the flow cell reactor for CO_2RR test.

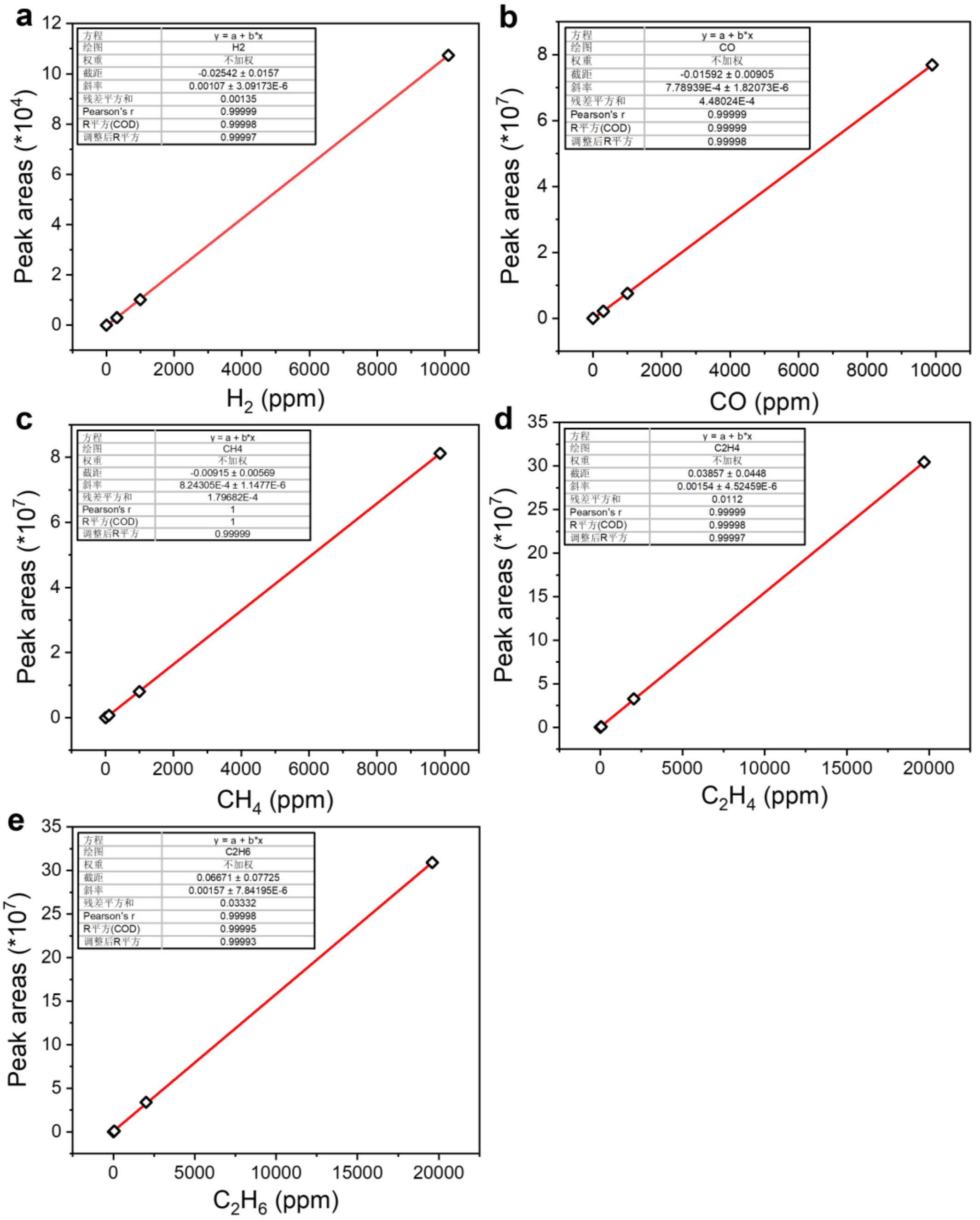

Figure S16. The method for quantitative analysis of gas products. The corresponding calibration curves for H_2 (a), CO (b), CH_4 (c), C_2H_4 (d), and C_2H_6 (e) components were determined by gas chromatography.

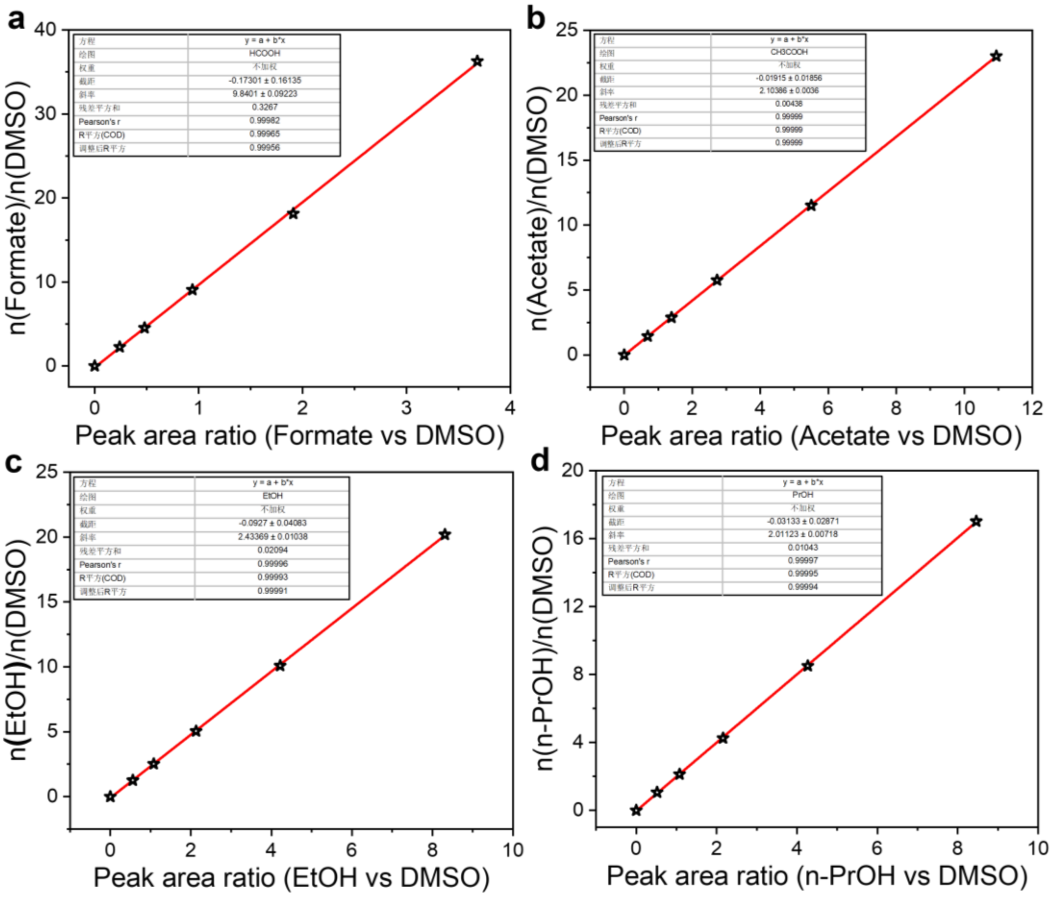

Figure S17. The method for quantitative analysis of liquid products. The standard curves for formate (a), acetate (b), ethanol (c), and n-propanol (d) ingredients were confirmed by means of ^{1}H NMR tests.

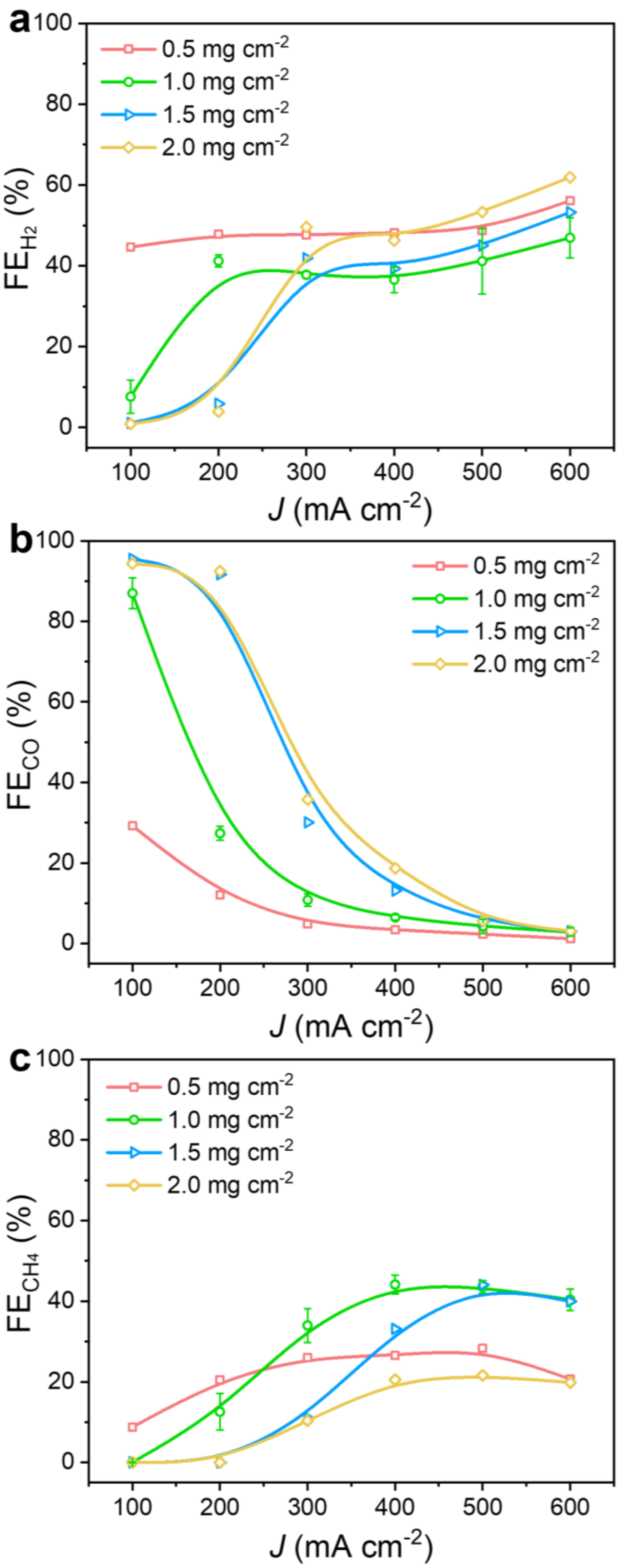

Figure S18. Faradaic efficiencies of H_2 (a), CO (b), and CH_4 (c) for Ni-N_3B_1/C with different catalyst loadings.

It can be found that Ni-N_3B_1/C shows higher CH_4 selectivity at the catalyst loading of 1.0 mg cm^{-2} than that of other loadings over the entire current density ranging. Therefore, the catalyst loading of 1.0 mg cm^{-2} was chosen for the subsequent electrochemical measurements.

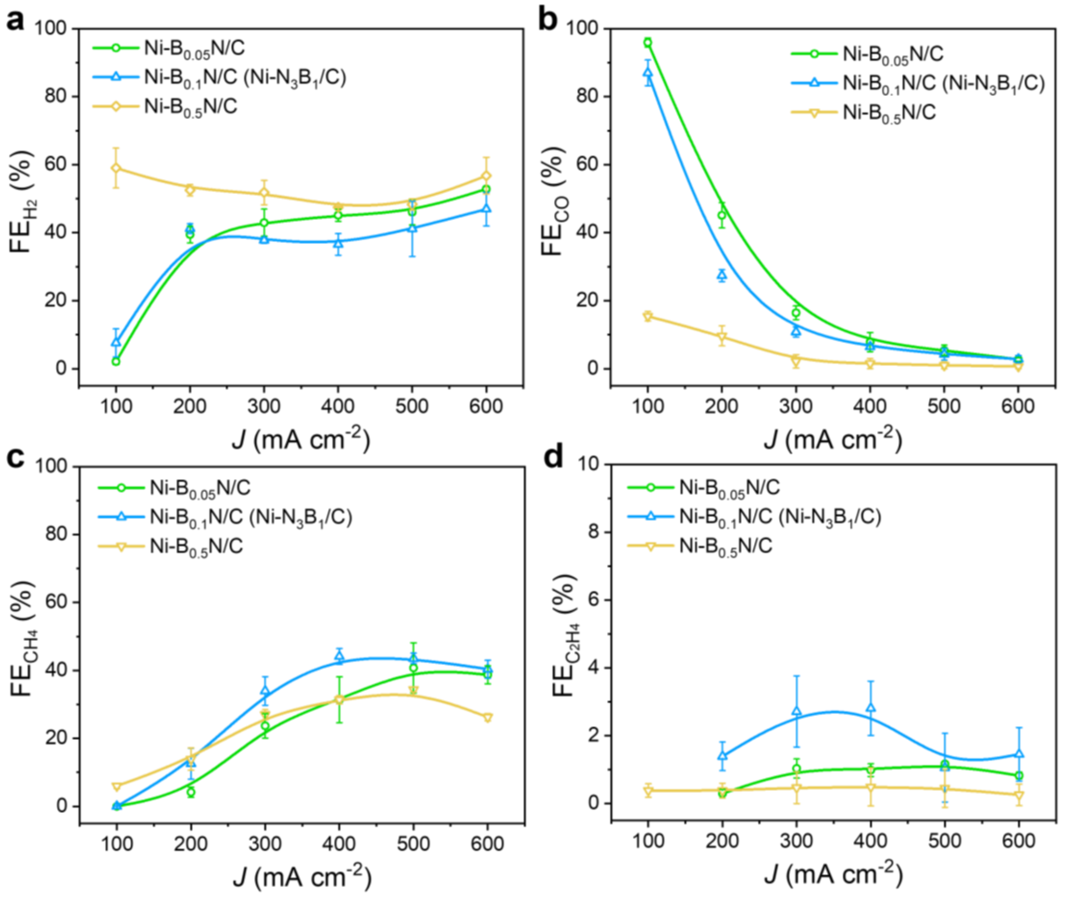

Figure S19. Faradaic efficiencies of H_2 (a), CO (b), CH_4 (c), and C_2H_4 (d) for Ni-BNC samples with different B contents.

To change the content of B, we adjusted the addition amount of boric acid to the precursor to 0.05 g, 0.1 g, and 0.5 g, respectively. The prepared samples were denoted as Ni-$B_{0.05}$NC, Ni-$B_{0.1}$NC, and Ni-$B_{0.5}$NC. As displayed by Figure S19, the quality of boric acid incorporation in the precursors exhibited a remarkable effect on the CO_2RR performance.

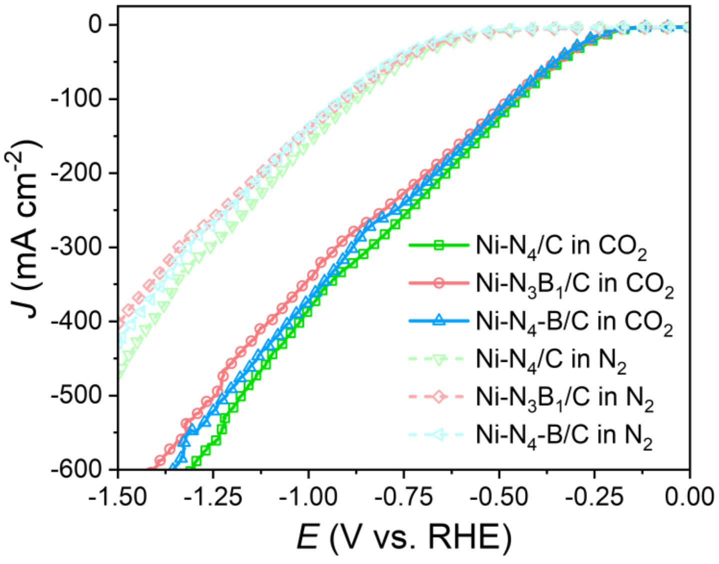

Figure S20. LSV curves of Ni-N_4/C, Ni-N_3B_1/C, and Ni-N_4-B/C in CO_2 and N_2 atmospheres. The measurement was conducted in 1 M KOH electrolyte with a scanning rate of 20 mV s^{-1}.

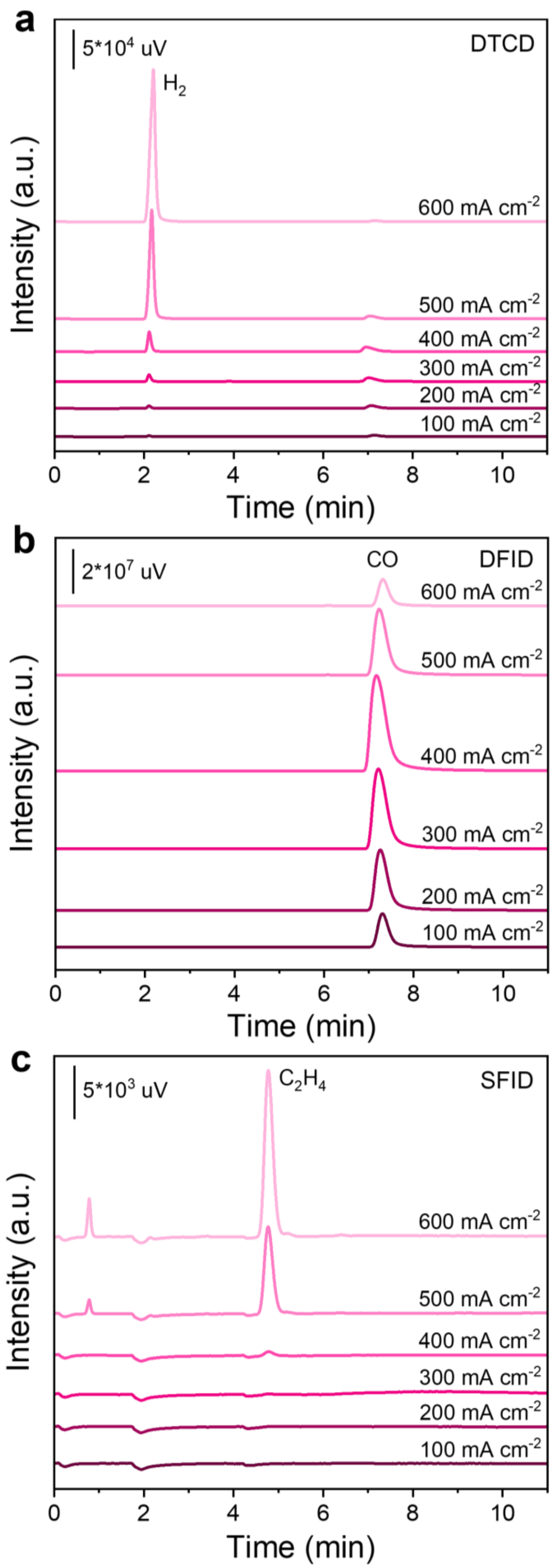

Supplementary Figure 21. The gas chromatography signals for Ni-N_4/C.

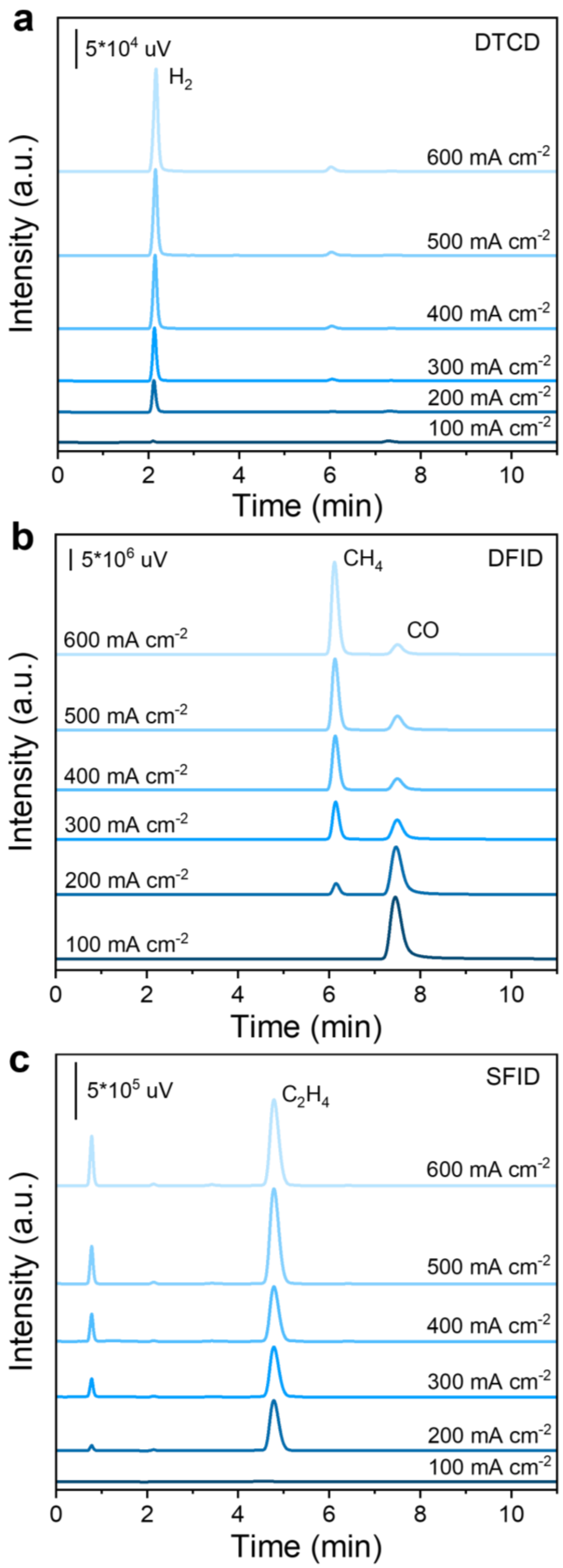

Figure S22. The gas chromatography signals for Ni-N_3B_1/C.

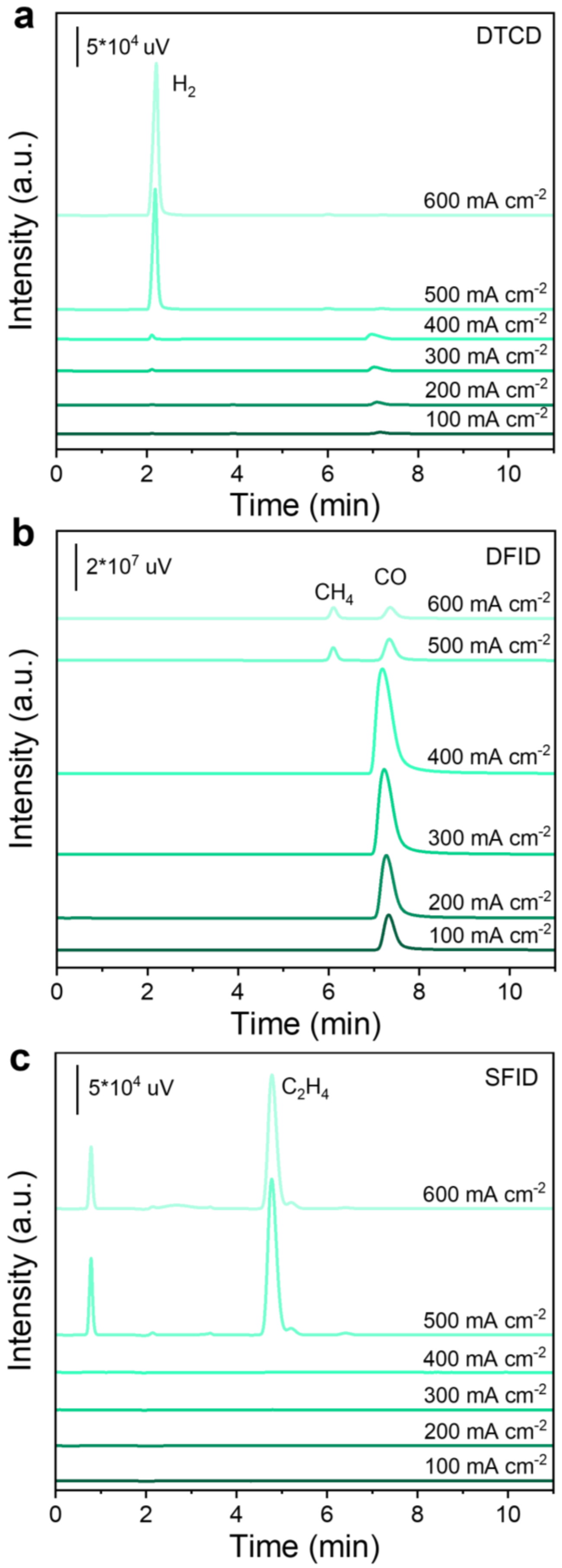

Figure S23. The gas chromatography signals for Ni-N_4-B/C.

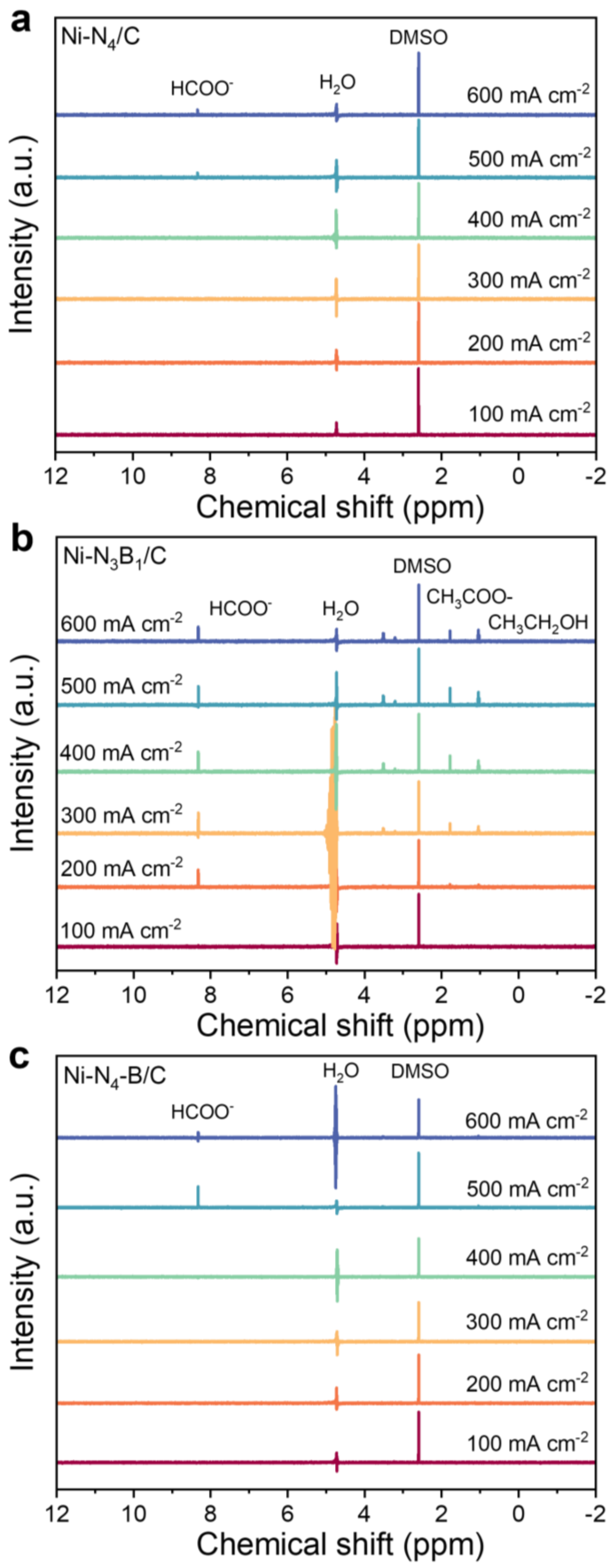

Figure S24. ^{1}H NMR spectra of Ni-N_4/C (a), Ni-N_3B_1/C (b), and Ni-N_4-B/C (c).

We collected the cathodic electrolyte after each current density to examine the liquid products. During the ^{1}H NMR measurements, DMSO was used as an internal standard for quantification of liquid products. Interestingly, formate, acetate, and ethanol were discerned in Ni-N_3B_1/C, while only formate could be observed in Ni-N_4/C and Ni-N_4-B/C, and the latter also exhibited a slight ethanol signal at high current densities.

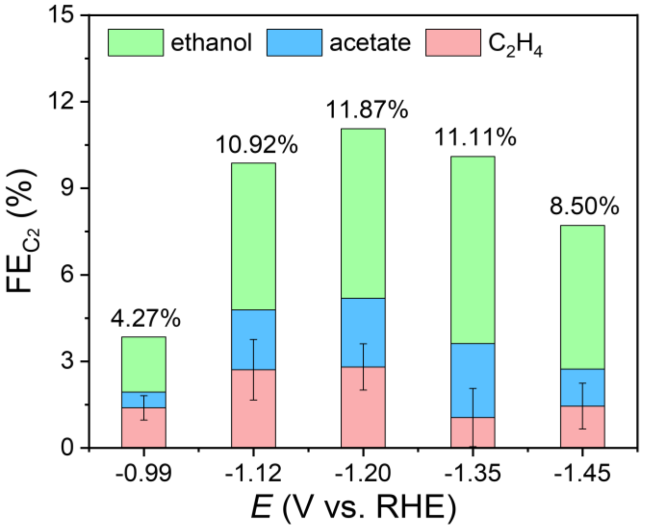

Figure S25. Faradaic efficiencies of C_2 products on Ni-N_3B_1/C.

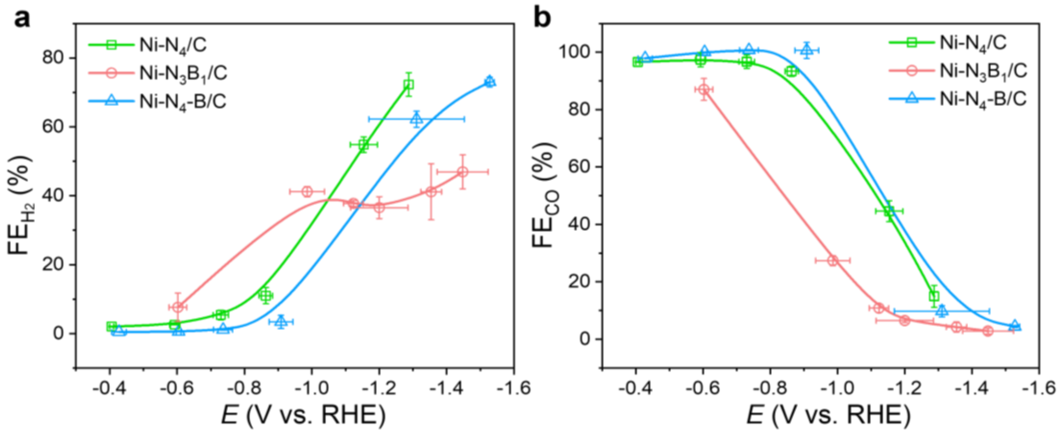

Figure S26. Faradaic efficiencies of H_2 (a) and CO (b) recorded at different applied potentials on Ni-N_4/C, Ni-N_3B_1/C, and Ni-N_4-B/C. Error bars are based on the mean ± standard deviation of three independent measurements.

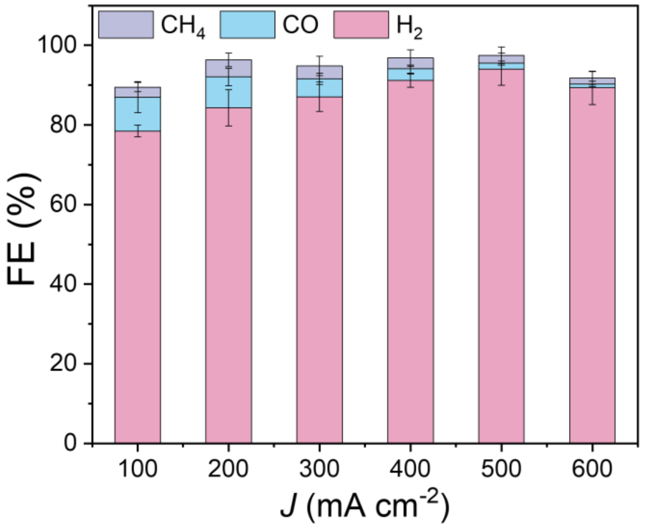

Figure S27. Faradaic efficiencies for various products on BN/C at different applied current densities. The product detected in significant quantities was H_2, with trivial CO and CH_4 generation. Error bars are based on the mean ± standard deviation of three independent measurements.

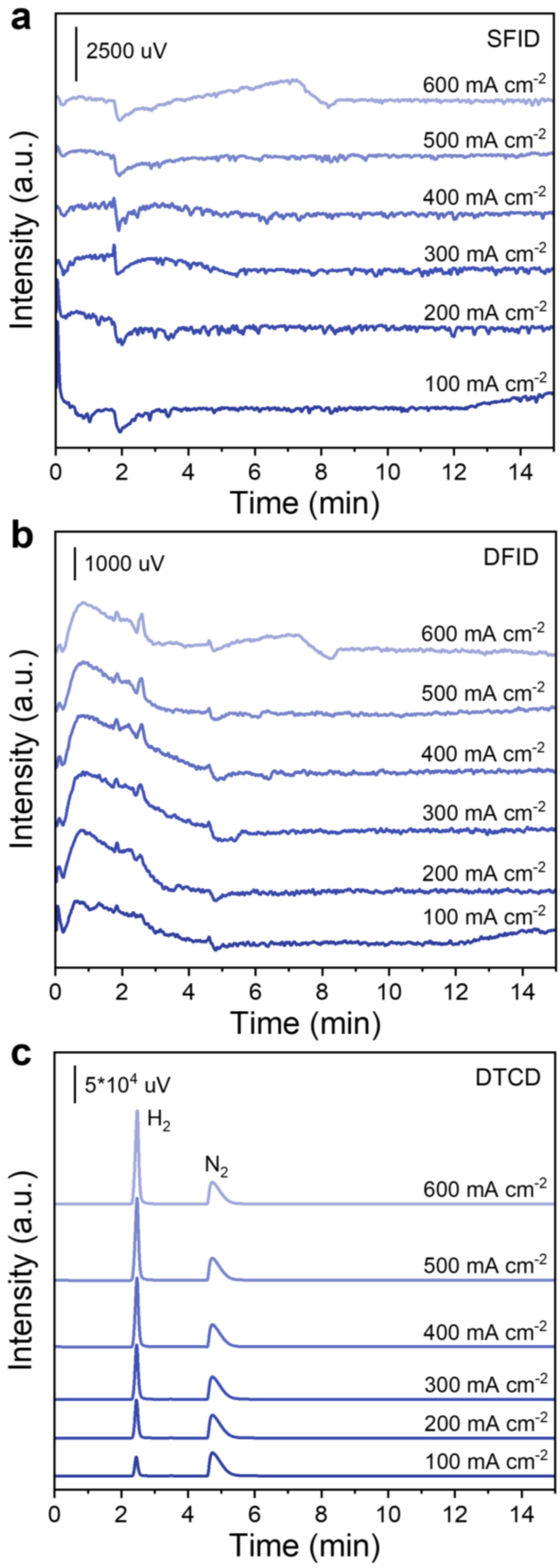

Figure S28. The gas chromatography signals for Ni-N_3B_1/C in N_2-saturated 1 M KOH electrolyte. Only the signal for H_2 could be observed across the entire investigated current densities, indicating the generation of CH_4 was derived from CO_2 molecules.

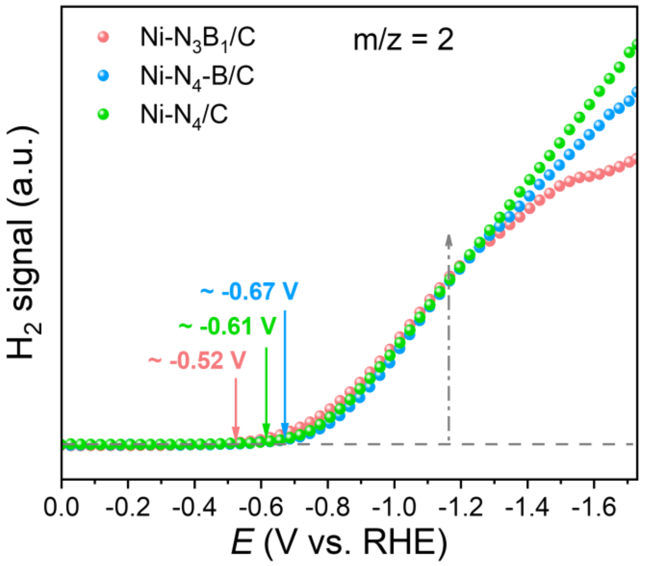

Figure S29. In situ DEMS test for H_2 production on Ni single-atom catalysts.

The onset potentials for Ni-N_4/C, Ni-N_3B_1/C, and Ni-N_4-B/C are −0.61 V, −0.52 V, and −0.67 V vs. RHE, respectively, which matched well with the electrochemical experimental results. Notably, Ni-N_3B_1/C exhibits the weakest H_2 signal at more negative potential compared with Ni-N_4/C and Ni-N_4-B/C. This is also coincident with the performance test, that is, Ni-N_3B_1/C possesses the lowest H_2 selectivity, especially at higher potentials.

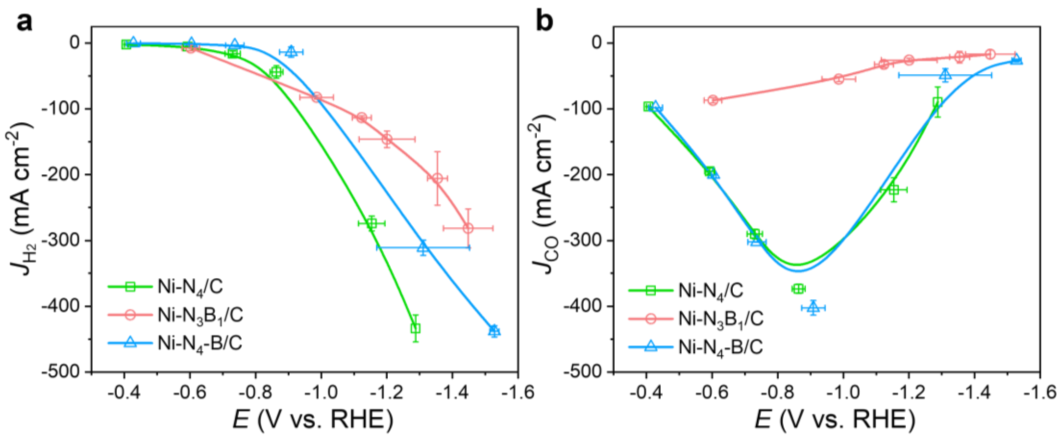

Figure S30. Partial current densities for H_2 (a) and CO (b) on various Ni single-atom catalysts. Error bars are based on the mean ± standard deviation of three independent measurements.

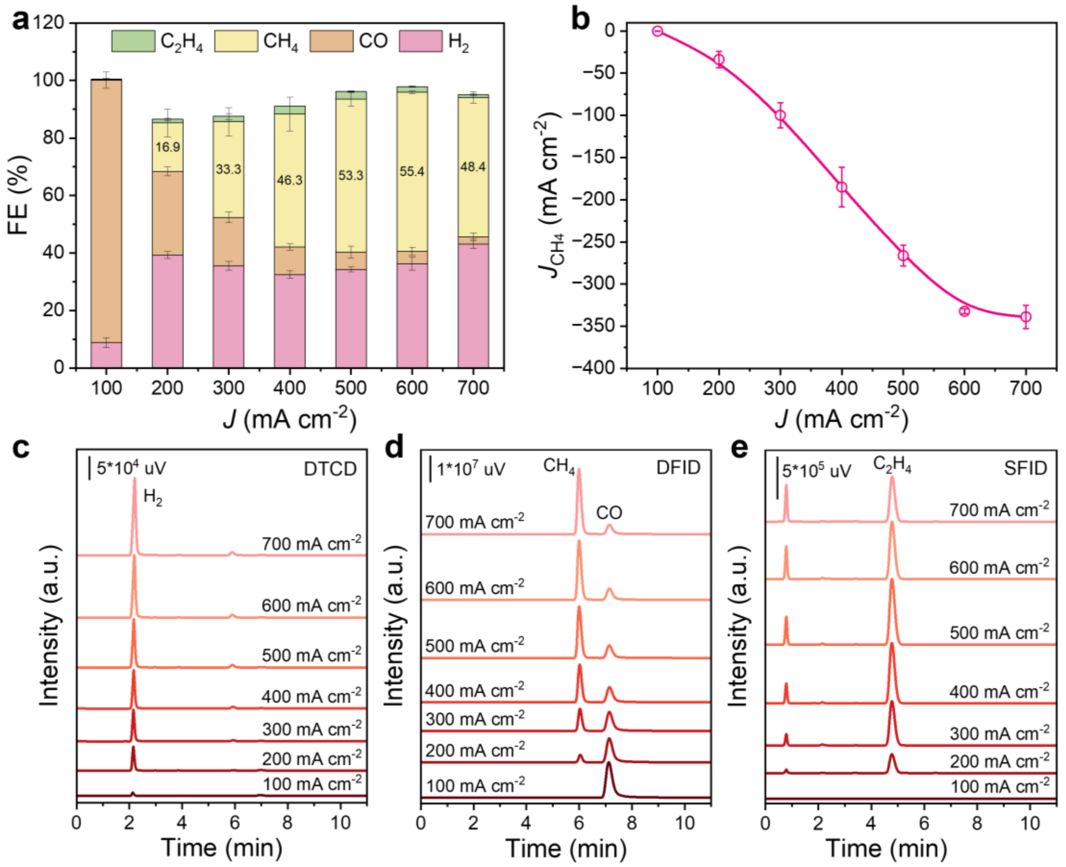

Figure S31. The distribution of gas phase products (a) and the partial current density of CH_4 (b) on Ni-N_3B_1/C with anion exchange membrane. The corresponding gas chromatography signals for H_2 (c), CH_4 and CO (d), and C_2H_4 (e).

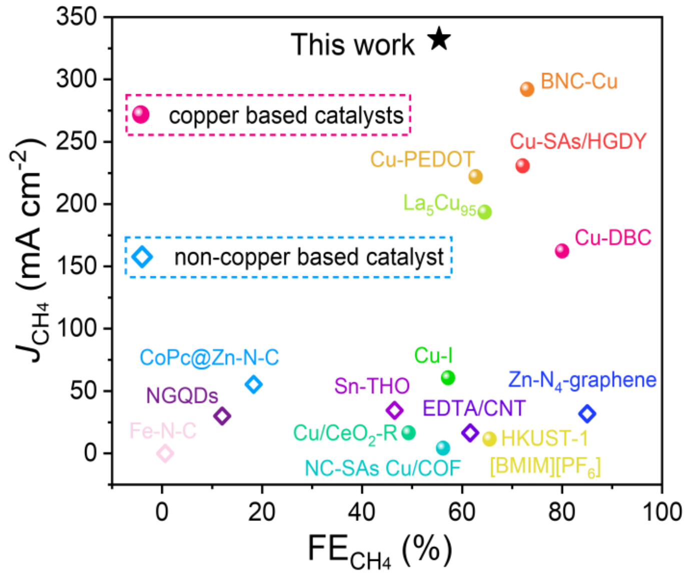

Figure S32. Contrasting the FEs and partial current density of CH_4 for Ni-N_3B_1/C and other reported advanced catalysts.

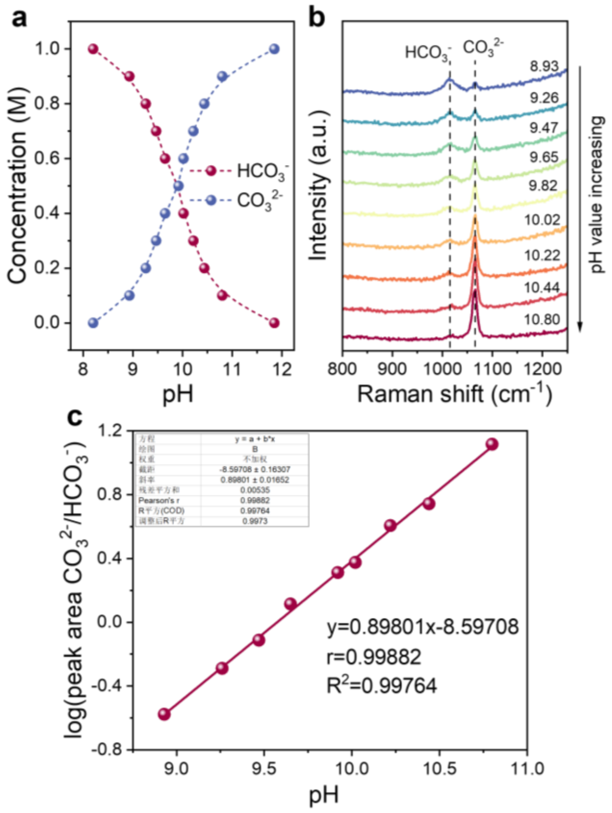

Figure S33. Local pH measurements during CO_2RR. (a) The theoretical pH of mixing solution containing different ration of $KHCO_3$ and K_2CO_3 totaling 1 M electrolyte concentration. (b) Raman spectra of the Ni-N_3B_1/C catalyst in solutions of various pH values (8.93~10.80). The peaks at ~1065 cm^{-1} and ~1015 cm^{-1} were assigned to CO_3^{2-} and HCO_3^{-}, respectively. (c) Calibration curves for local pH using the CO_3^{2-} and HCO_3^{-} peak area ratio.

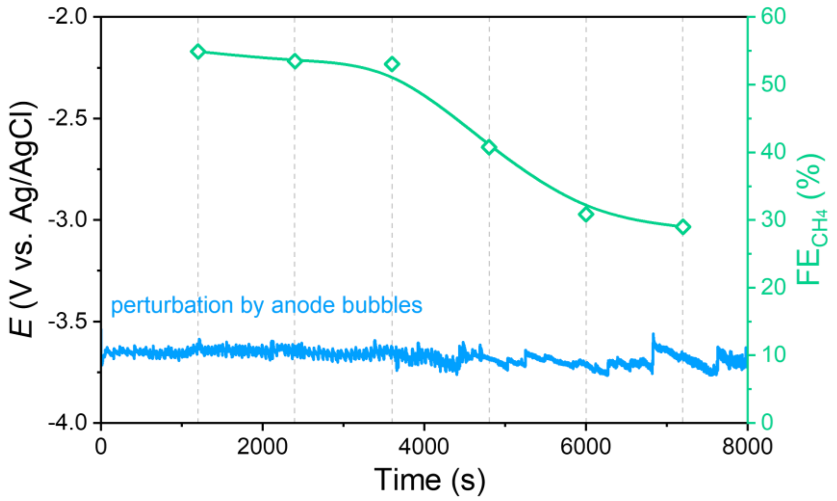

Figure S34. Stability test of the Ni-N_3B_1/C catalyst at 500 mA cm^{-2} in 1 M KOH electrolyte. The potential could be maintained at −3.65 V vs. Ag/AgCl during the long-term operation whereas the FE_{CH_4} sharply decreased after 1 h.

We speculated that the hydrophobic layer of the gas diffusion electrode (GDE) might be destroyed during operation at such high current density, which concentrated more water molecules and impeded the CO_2 diffusion, thus resulting in reduced FE_{CH_4} and enhanced FE_{H_2}. To better guarantee the CH_4 activity, decreasing the applied current density (at the expense of FE_{CH_4}) or improving the hydrophobicity of GDE is warranted.

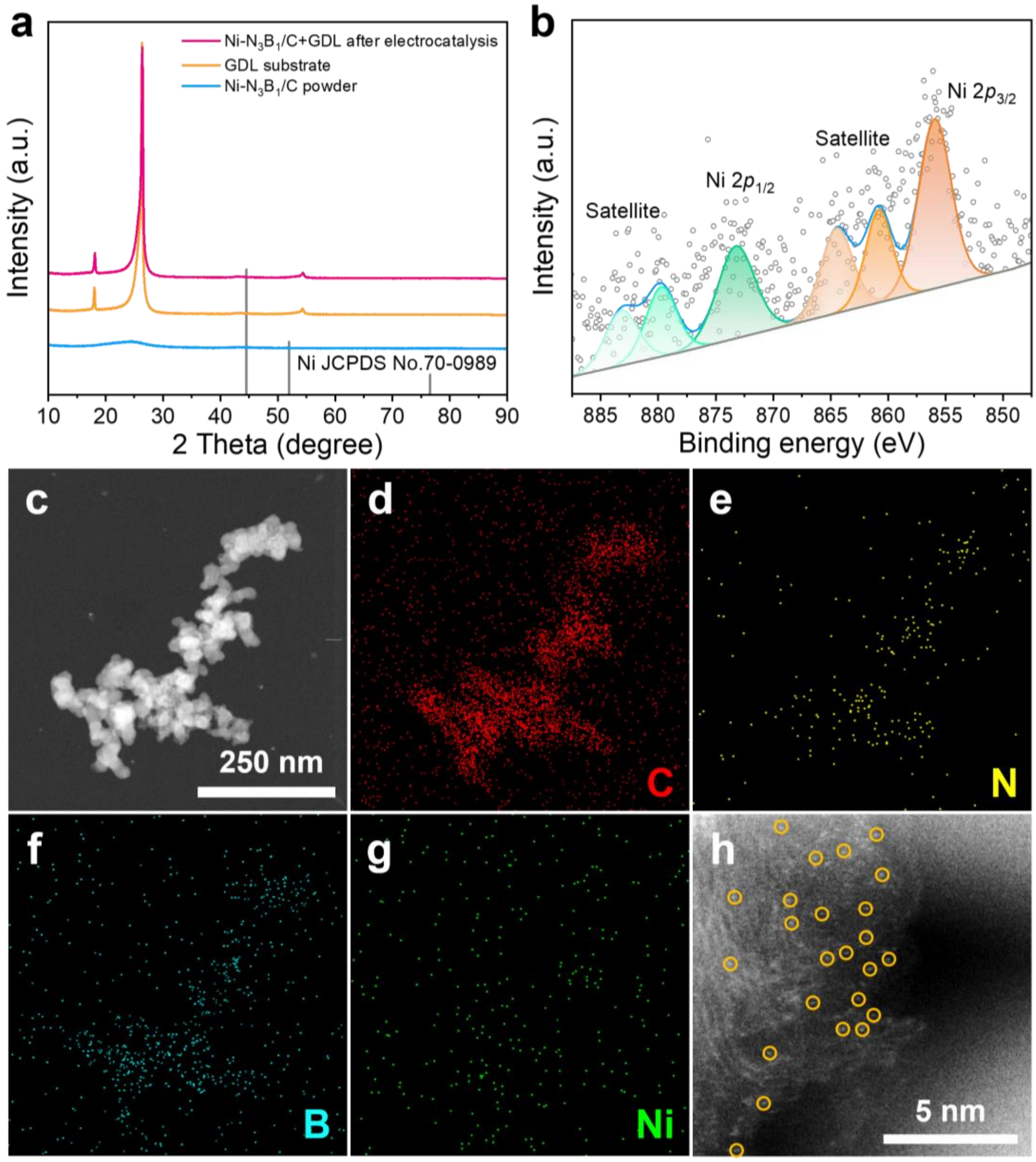

Figure S35. The structure and morphology characterization of Ni-N_3B_1/C after electrolysis. (a) XRD patterns, (b) high-resolution Ni 2*p* spectra, (c-g) HRTEM and corresponding element mapping images, and (h) Aberration-corrected HAADF-STEM image.

The XRD patterns indicate the absence of peaks corresponding to Ni metal, with only signals from the substrate observed. This suggests that there was no aggregation of Ni atoms throughout the electrolysis process. The Ni 2*p* spectra exhibit characteristic peaks for Ni^{2+}, consistent with pre-electrolysis results. Complementary HRTEM and element mapping, along with aberration-corrected HAADF-STEM image, further confirm the preservation of atomic structure in the Ni-N_3B_1/C catalyst. Overall, these analyses demonstrate the exceptional stability of the Ni-N_3B_1/C catalyst under electrolysis conditions.

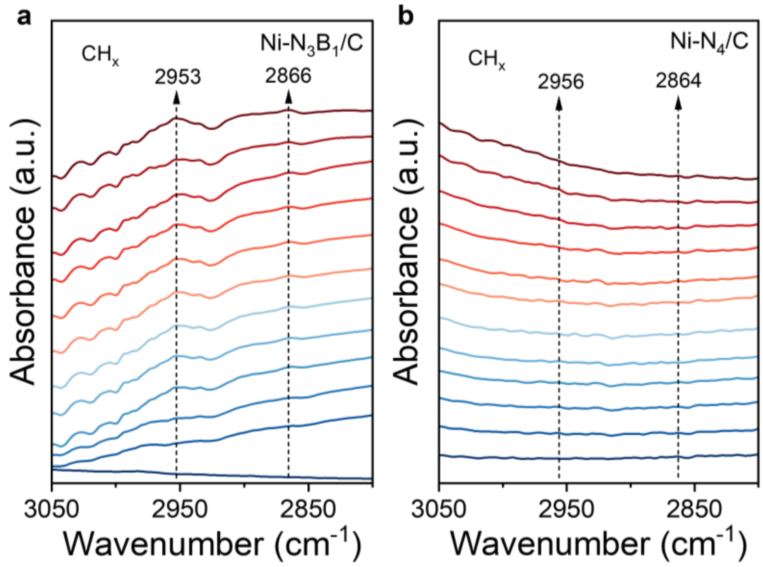

Figure S36. In situ ATR-SEIRAS spectra about CH_x signals at various potentials in a CO_2-saturated 0.1 M $KHCO_3$ electrolyte over the Ni-N_3B_1/C (a) and Ni-N_4/C (b) catalysts.

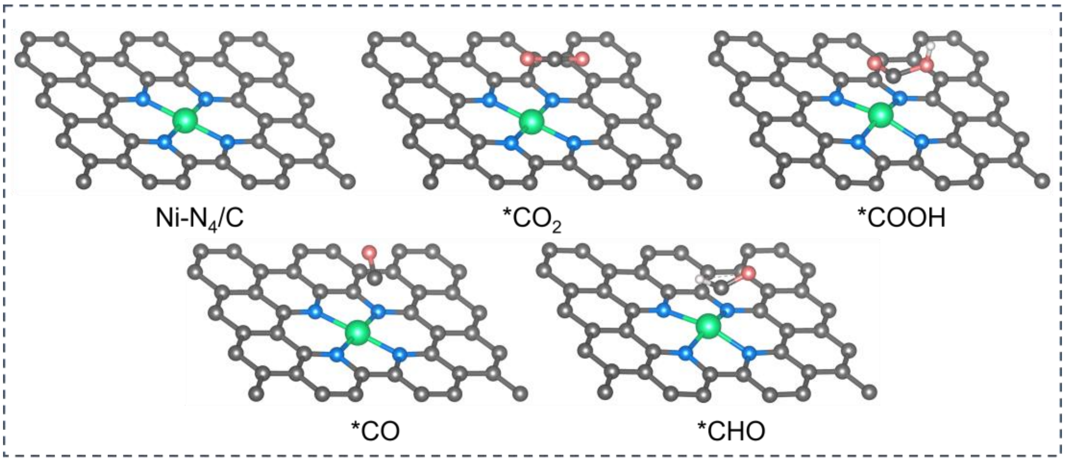

Figure S37. The optimal adsorption configurations of *CO_2, *COOH, *CO, and *CHO on Ni-N_4/C (Gray for C, blue for N, green for Ni, red for O, and white for H).

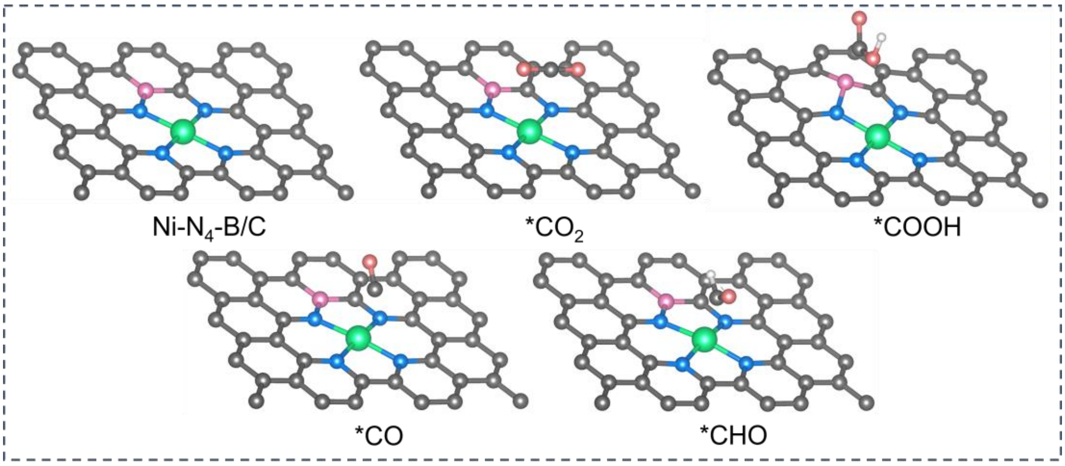

Figure S38. The optimal adsorption configurations of *CO_2, *COOH, *CO, and *CHO on Ni-N_4-B/C (Gray for C, blue for N, green for Ni, pink for B, red for O, and white for H).

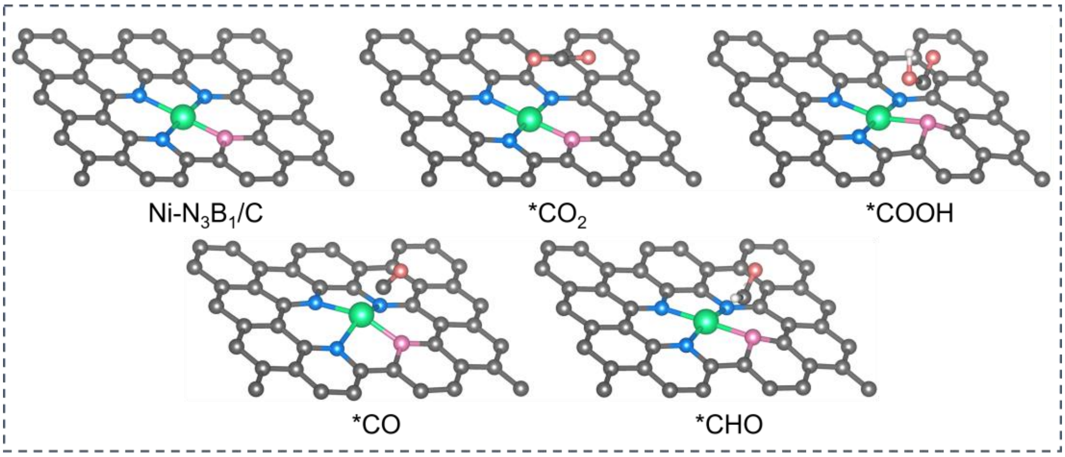

Figure S39. The optimal adsorption configurations of *CO_2, *COOH, *CO, and *CHO on Ni-N_3B_1/C (Gray for C, blue for N, green for Ni, pink for B, red for O, and white for H).

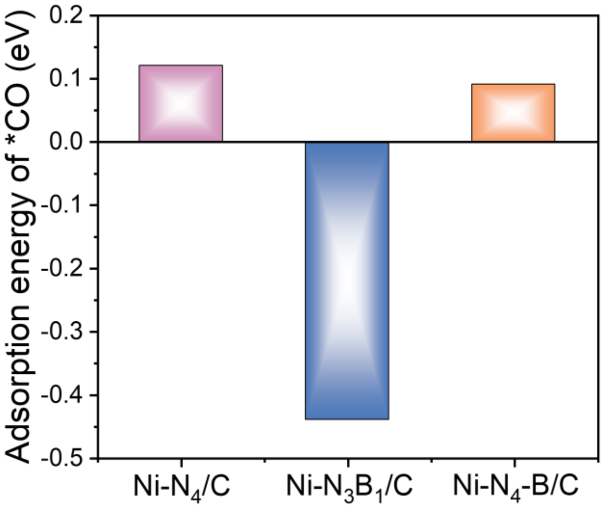

Figure S40. The adsorption energy of *CO intermediates on these three catalysts.

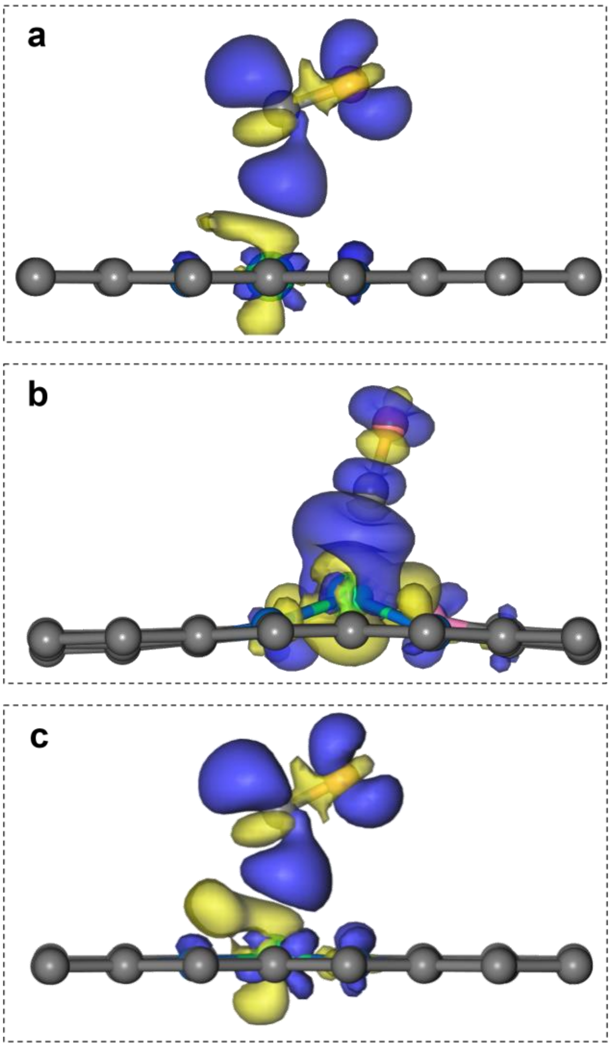

Figure S41. Differential charge density distribution of Ni-N_4/C (a), Ni-N_3B_1/C (b), and Ni-N_4-B/C (c) with *CO intermediate adsorption. Blue and yellow isosurfaces show electron accumulation and depletion. To enhance the aesthetic appeal of the display, the isosurface values of Ni-N_4/C and Ni-N_4-B/C were set to 0.005 e Å^{-3} and the isosurface value of Ni-N_3B_1/C was set to 0.02 e Å^{-3}.

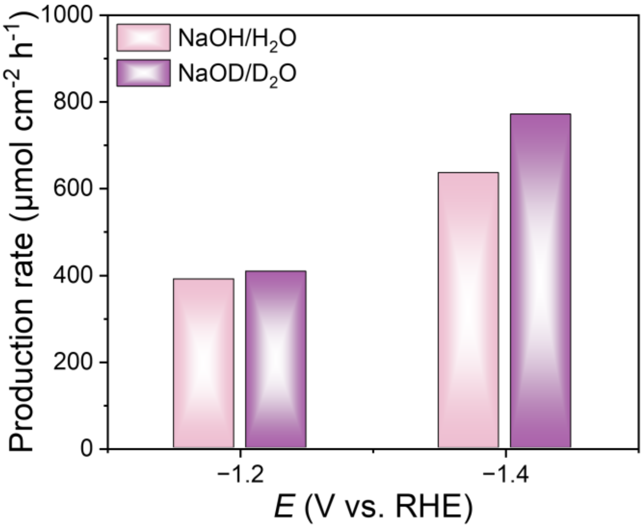

Figure S42. The production rate of CO_2 electroreduction to CH_4 on the Ni-N_3B_1/C catalyst at −1.2 V and −1.4 V vs. RHE.

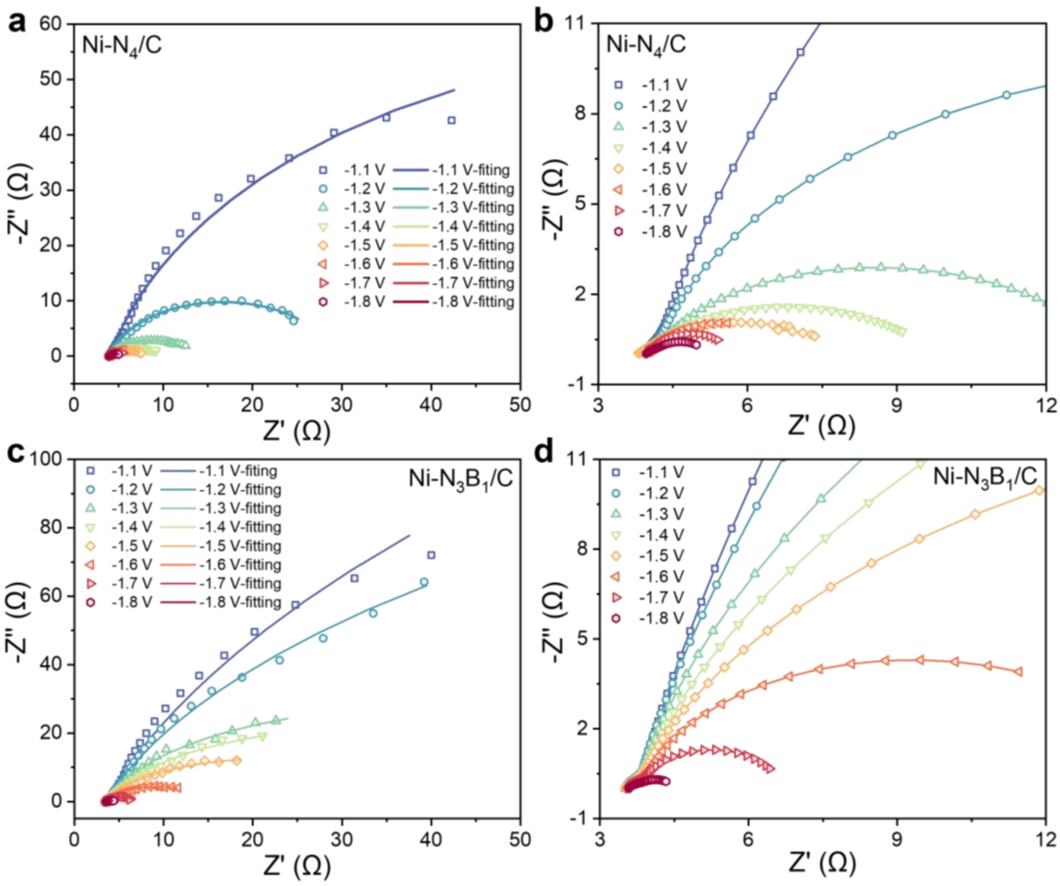

Figure S43. EIS plots of Ni-N_4/C (a, b) and Ni-N_3B_1/C (c, d) under different applied potentials.

Table S1. EXAFS curve fitting parameters at the Ni *K*-edge for various samples (S_0^2 = 0.86).

Sample	Path	C.N.	R(Å)	σ^2(×10^{-3} Å^2)	ΔE_0(eV)	R factor
Ni-N_4/C	Ni-N	3.9 ±0.6	1.85 ±0.01	6.9 ±1.6	−6.6 ±0.7	0.01
	Ni-C (second-shell)	3.9 ±2.6	3.04 ±0.02	7.5 ±7.3		
Ni-N_3B_1/C	Ni-N	3.0 ±0.5	1.90 ±0.02	8.4 ±8.5	−4.6 ±3.3	0.02
	Ni-B	1.0 ±0.3	2.17 ±0.02	4.6 ±0.6		
	Ni-C (second-shell)	2.4 ±0.5	2.79 ±0.03	6.3 ±1.5		
Ni-N_4-B/C	Ni-N	3.5 ±0.6	1.89 ±0.02	5.6 ±2.2	−2.1 ±3.2	0.02
	Ni-B/C (second-shell)	2.8 ±0.5	2.86 ±0.02	4.1 ±1.2		

S_0^2: the amplitude reduction factor; C.N.: coordination number; R: bond distance; σ^2: Debye-Waller factors; ΔE_0: the inner potential correction; R factor: goodness of fit.

* This value was fixed during EXAFS fitting, based on the known structure of Ni foil.

Table S2. Comparison of CO_2RR for CH_4 production between this work and other advanced catalysts.

**Catalyst**	**Electrolyte**	**FE_{CH4} (%)**	**J_{CH4} (mA cm^{-2})**	**E (V vs. RHE)**	**Refs**
Cu-DBC	1 M KOH	80	−162.3	−0.9	[7]
Cu-SAs/HGDY	1 M KOH	72.1	−230.7	−1.1	[8]
BNC-Cu	0.5 M $KHCO_3$	73	−292	−1.46	[9]
CuPEDOT	1 M KOH	62.7	−222	−1.58	[10]
HKUST-1/[BMIM][PF_6] hybrid	0.1 M $KHCO_3$	65.5	−11.5	−1.13	[11]
La_5Cu_{95}	1 M KOH	64.5	−193.5	−1.72	[12]
Cu-I	1 M KOH	57.2	−60.7	−1.08	[13]
Cu/CeO_2-R	0.1 M $KHCO_3$	49.3	−16.5	−1.6	[14]
NC-SA Cu/COF	0.1 M $NaHCO_3$	56.2	−4.2	−1.26	[15]
CoPc@Zn-N-C	1 M KOH	18.3	−44.3	−1.24	[16]
Zn-N_4-graphene	1 M $KHCO_3$	85	−31.8	−1.8 (vs. SCE)	[17]
EDTA/CNT	0.5 M $KHCO_3$	61.6	−16.5	−1.3	[18]
Sn-THO	0.1 M $KHCO_3$	46.5	−34.5	−1.6	[19]
NGQDs	1 M KOH	12	−30	−1.05	[20]
Fe-N-C	0.05 M K_3PO_4 + 0.05 M H_3PO_4	0.6	−0.1	−0.6	[21]
**Ni-N_3B_1/C**	**1 M KOH**	**55.4**	**−332.4**	**−1.51**	**This work**

Table S3. The bond length analysis after CO adsorption on Ni-N_4/C, Ni-N_4-B/C, and Ni-N_3B_1/C.

**Samples**	**Ni-C**	**C-O**
Ni-N_4/C-CO	2.629 Å	1.148 Å
Ni-N_4-B/C-CO	2.402 Å	1.151 Å
Ni-N_3B_1/C-CO	1.758 Å	1.160 Å

Table S4. The calculated Mulliken population analysis after CO adsorption on Ni-N_4/C, Ni-N_4-B/C, and Ni-N_3B_1/C.

**Samples**	**Ni**	**B**	**CO**
Ni-N_4/C-CO	1.23 e	−	−0.07 e
Ni-N_4-B/C-CO	1.24 e	0.57 e	−0.08 e
Ni-N_3B_1/C-CO	1.27 e	0.15 e	−0.17 e

References

1. Min Y, Zhou X, Chen J-J *et al.* Integrating single-cobalt-site and electric field of boron nitride in dechlorination electrocatalysts by bioinspired design. *Nat Commun* 2021; **12**: 303.
2. Milman V, Winkler B, White JA *et al.* Electronic structure, properties, and phase stability of inorganic crystals: A pseudopotential plane-wave study. *Int J Quantum Chem* 2000; **77**: 895-910.
3. Perdew JP, Burke K, Ernzerhof M. Generalized gradient approximation made simple. *Phys Rev Lett* 1996; **77**: 3865-3868.
4. Perdew JP, Ruzsinszky A, Csonka GI *et al.* Restoring the density-gradient expansion for exchange in solids and surfaces. *Phys Rev Lett* 2008; **100**: 136406.
5. Head JD, Zerner MC. A broyden-fletcher-goldfarb-shanno optimization procedure for molecular geometries. *Chem Phys Lett* 1985; **122**: 264-270.
6. Nørskov JK, Rossmeisl J, Logadottir A *et al.* Origin of the overpotential for oxygen reduction at a fuel-cell cathode. *The Journal of Physical Chemistry B* 2004; **108**: 17886-17892.
7. Zhang Y, Dong L-Z, Li S *et al.* Coordination environment dependent selectivity of single-site-Cu enriched crystalline porous catalysts in CO_2 reduction to CH_4. *Nat Commun* 2021; **12**: 6390.

8. Zhao P, Jiang H, Shen H *et al.* Construction of low-coordination Cu-C_2 single-atoms electrocatalyst facilitating the efficient electrochemical CO_2 reduction to methane. *Angew Chem Int Ed* 2023; **62**: e202314121.
9. Dai Y, Li H, Wang C *et al.* Manipulating local coordination of copper single atom catalyst enables efficient CO_2-to-CH_4 conversion. *Nat Commun* 2023; **14**: 3382.
10. Chen X, Jia S, Chen C *et al.* Highly stable layered coordination polymer electrocatalyst toward efficient CO_2-to-CH_4 conversion. *Adv Mater* 2024; **36**: 2310273.
11. Delmo EP, Wang Y, Wang J *et al.* Metal organic framework-ionic liquid hybrid catalysts for the selective electrochemical reduction of CO_2 to CH_4. *Chin J Catal* 2022; **43**: 1687-1696.
12. Zhao J, Zhang P, Yuan T *et al.* Modulation of *CH_xO adsorption to facilitate electrocatalytic reduction of CO_2 to CH_4 over Cu-based catalysts. *J Am Chem Soc* 2023; **145**: 6622-6627.
13. Zhang Y, Zhou Q, Qiu Z-F *et al.* Tailoring coordination microenvironment of Cu(I) in metal-organic frameworks for enhancing electroreduction of CO_2 to CH_4. *Adv Funct Mater* 2022; **32**: 2203677.
14. Xue L, Zhang C, Wu J *et al.* Unveiling the reaction pathway on Cu/CeO_2 catalyst for electrocatalytic CO_2 reduction to CH_4. *Appl Catal, B* 2022; **304**: 120951.
15. Zhao Q, Wang Y, Li M *et al.* Organic frameworks confined Cu single atoms and nanoclusters for tandem electrocatalytic CO_2 reduction to methane. *SmartMat* 2022; **3**: 183-193.
16. Lin L, Liu T, Xiao J *et al.* Enhancing CO_2 electroreduction to methane with a cobalt phthalocyanine and zinc-nitrogen-carbon tandem catalyst. *Angew Chem Int Ed* 2020; **59**: 22408-22413.
17. Han L, Song S, Liu M *et al.* Stable and efficient single-atom Zn catalyst for CO_2 reduction to CH_4. *J Am Chem Soc* 2020; **142**: 12563-12567.
18. Huang M, Gong S, Wang C *et al.* Lewis-basic edta as a highly active molecular electrocatalyst for CO_2 reduction to CH_4. *Angew Chem Int Ed* 2021; **60**: 23002-23009.
19. Zhao Z-H, Huang J-R, Liao P-Q *et al.* Isolated tin(IV) active sites for highly efficient electroreduction of CO_2 to CH_4 in neutral aqueous solution. *Angew Chem Int Ed* 2023; **62**: e202301767.
20. Wu J, Ma S, Sun J *et al.* A metal-free electrocatalyst for carbon dioxide reduction to multi-carbon hydrocarbons and oxygenates. *Nat Commun* 2016; **7**: 13869.
21. Ju W, Bagger A, Wang X *et al.* Unraveling mechanistic reaction pathways of the electrochemical CO_2 reduction on Fe–N–C single-site catalysts. *ACS Energy Lett* 2019; **4**: 1663-1671.
